# Tissue‐Engineered Disease Modeling of Lymphangioleiomyomatosis Exposes a Therapeutic Vulnerability to HDAC Inhibition

**DOI:** 10.1002/advs.202302611

**Published:** 2023-07-03

**Authors:** Adam Pietrobon, Julien Yockell‐Lelièvre, Nicole Melong, Laura J. Smith, Sean P. Delaney, Nadine Azzam, Chang Xue, Nishanth Merwin, Eric Lian, Alberto Camacho‐Magallanes, Carole Doré, Gabriel Musso, Lisa M. Julian, Arnold S. Kristof, Roger Y. Tam, Jason N. Berman, Molly S. Shoichet, William L. Stanford

**Affiliations:** ^1^ The Sprott Centre for Stem Cell Research Regenerative Medicine Program Ottawa Hospital Research Institute Ottawa K1Y 4E9 Canada; ^2^ Department of Cellular and Molecular Medicine University of Ottawa Ottawa K1N 6N5 Canada; ^3^ Ottawa Institute of Systems Biology Ottawa K1H 8M5 Canada; ^4^ Department of Pediatrics CHEO Research Institute Ottawa K1H 5B2 Canada; ^5^ Department of Chemical Engineering and Applied Chemistry University of Toronto Toronto M5S 3E5 Canada; ^6^ Institute for Biomaterials and Biomedical Engineering University of Toronto Toronto M5S 3G9 Canada; ^7^ The Donnelly Centre for Cellular and Biomolecular Research Toronto M5S 3E1 Canada; ^8^ BioSymetrics Inc. Toronto M5T 1X5 Canada; ^9^ Centre for Cell Biology Development and Disease Department of Biological Sciences Simon Fraser University Burnaby V5A 1S6 Canada; ^10^ Meakins‐Christie Laboratories and Translational Research in Respiratory Diseases Program Research Institute of the McGill University Health Centre Faculty of Medicine Departments of Medicine and Critical Care Montreal H4A 3J1 Canada; ^11^ Centre for Biologics Evaluation Biologic and Radiopharmaceutical Drugs Directorate Health Canada Ottawa K1Y 4X2 Canada; ^12^ Department of Chemistry University of Toronto Toronto M5S 3H6 Canada

**Keywords:** 3D drug screen, biomimetic hydrogel culture, HDAC inhibition, lymphangioleiomyomatosis, mTORC1, therapeutics development, zebrafish

## Abstract

Lymphangioleiomyomatosis (LAM) is a rare disease involving cystic lung destruction by invasive LAM cells. These cells harbor loss‐of‐function mutations in *TSC2*, conferring hyperactive mTORC1 signaling. Here, tissue engineering tools are employed to model LAM and identify new therapeutic candidates. Biomimetic hydrogel culture of LAM cells is found to recapitulate the molecular and phenotypic characteristics of human disease more faithfully than culture on plastic. A 3D drug screen is conducted, identifying histone deacetylase (HDAC) inhibitors as anti‐invasive agents that are also selectively cytotoxic toward *TSC2^−/−^* cells. The anti‐invasive effects of HDAC inhibitors are independent of genotype, while selective cell death is mTORC1‐dependent and mediated by apoptosis. Genotype‐selective cytotoxicity is seen exclusively in hydrogel culture due to potentiated differential mTORC1 signaling, a feature that is abrogated in cell culture on plastic. Importantly, HDAC inhibitors block invasion and selectively eradicate LAM cells in vivo in zebrafish xenografts. These findings demonstrate that tissue‐engineered disease modeling exposes a physiologically relevant therapeutic vulnerability that would be otherwise missed by conventional culture on plastic. This work substantiates HDAC inhibitors as possible therapeutic candidates for the treatment of patients with LAM and requires further study.

## Graphical abstract

3D hydrogel culture renders *TSC2^−/−^* lymphangioleiomyomatosis (LAM) cells vulnerable to histone deacetylase (HDAC) inhibitor treatment in an mTORC1‐dependent manner. The genotype‐selective cytotoxicity of HDAC inhibitors is abrogated in conventional 2D culture on plastic. HDAC inhibitors are found to block invasion and selectively eradicate *TSC2^−/−^* LAM cells in an in vivo zebrafish xenograft assay.

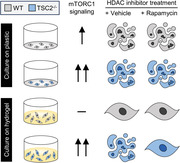

## 1 Introduction

Lymphangioleiomyomatosis (LAM) is a cystic lung disease predominately affecting women, at a prevalence of 1 to 10 per million.^[^ ^1^ ^]^ LAM can occur sporadically or in association with the multisystem tumor‐forming disorder, tuberous sclerosis complex (TSC).^[^ ^2^ ^]^ The pulmonary histopathology is characterized by microscopic nodules consisting of immature smooth muscle‐like cells that express markers of neural crest lineages.^[^ ^3^ ^]^ These invading cells digest the lung parenchyma, forming cystic lesions that lead to progressive respiratory decline and fatality if untreated.^[^ ^4^, ^5^, ^6^ ^]^ The molecular etiology of LAM involves loss‐of‐function mutations in the endogenous mTORC1 suppressor *TSC2*, thereby inducing hyperactivation of mTORC1 anabolic and tumorigenic signaling.^[^ ^7^ ^]^ The allosteric mTORC1 inhibitor rapamycin (clinically, sirolimus) slows disease progression and improves symptomatology.^[^ ^8^, ^9^, ^10^, ^11^ ^]^ While clinical approval of rapamycin by the U.S. Food and Drug Administration in 2015 has led to a dramatic new frontier in the LAM therapeutic landscape, significant limitations exist. A subset of patients does not respond to treatment, and rapamycin is invariably cytostatic, with rapid disease progression upon treatment withdrawal.^[^ ^11^, ^12^ ^]^ There is a critical need to discover novel treatment strategies which block the invasive phenotype and/or eradicate neoplastic LAM cells.

A key step in the pathway to therapeutic development is the effective modeling of disease characteristics. In this domain, LAM has remained a challenge. Cultures of cells derived from human pulmonary LAM lesions grow as a heterogenous mixture with rapid exhaustion of *TSC2^−/−^* cells, prohibiting the establishment of clonal primary cell lines.^[^ ^13^ ^]^ While a genome engineering strategy would seem straightforward for this monogenic disease, the cell‐of‐origin of LAM remains unknown, begging the question of which cell type to engineer. While we have demonstrated that *TSC2^−/−^* human pluripotent stem cell‐derived neural crest cells model several phenotypic features of LAM,^[^ ^14^ ^]^ neural crest cells consist of a diverse and plastic population that are not readily scalable for drug screening purposes. Animal models of LAM have been comparably challenging to establish, and none to date have recapitulated pathognomonic features such as histological premelanosome protein (PMEL) positivity and concomitant elevated serum levels of vascular endothelial growth factor D (VEGF‐D).^[^ ^15^ ^]^

A critical consideration in disease modeling is the contribution of the extracellular matrix (ECM) to disease biology. Water‐swollen networks of polymers termed hydrogels have arisen as effective tools for mimicking salient elements of the native ECM while exhibiting mechanics similar to many soft tissues.^[^ ^16^ ^]^ Hydrogels can be broadly classified as either natural, synthetic, or hybrid materials. One such hybrid scaffold is hyaluronic acid, a naturally sourced material that can be readily modified to independently tune ECM features of interest, such as elasticity, stiffness, and viscosity.^[^ ^17^ ^]^ A viscoelastic hydrogel with a derivatized hyaluronic acid backbone has been shown to permit the study of invasive properties of LAM cellular models in 3D culture.^[^ ^18^ ^]^

In recent years, there has been a resurgence of interest in phenotype‐based screens for drug discovery compared to target‐based approaches.^[^ ^19^ ^]^ An analysis of therapeutics approved between 1999 and 2008 revealed that 62% first‐in‐class drugs were discovered by phenotype‐based screens, despite the fact that such screens represented only a small subset of the overall total.^[^ ^20^ ^]^ The apparent superiority of phenotype‐based approaches may in part arise from the ability to identify compounds which exhibit a therapeutic effect by modulating multiple targets simultaneously.^[^ ^19^ ^]^ In addition, phenotypic drug screens can be multiplexed with counterscreening, ensuring candidate therapeutics do not also confer undesirable side effects, such as physiological toxicity. In the context of LAM, a monogenic disease, this counterscreening takes shape by directly comparing *TSC2^−/−^* cells against matched wild type (WT) controls. Here, we employ isogenic *TSC2^−/−^* and WT LAM cell models to study mTORC1‐dependent drug responses and cellular behaviors, comparing between standard 2D culture on plastic versus 3D hydrogel culture. We subsequently assess promising drug candidates with an in vivo zebrafish xenograft assay.

## 2 Results

### 2.1 Stem Cell‐Derived Models Exhibit Features of LAM, Independent of Genotype

**Figure 1:**
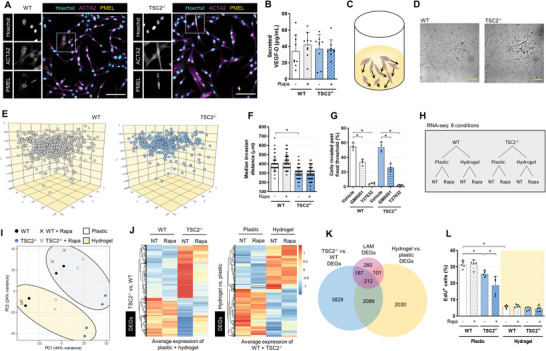
Hydrogel culture of stem cell‐derived disease models exhibits features of LAM. A) Representative immunofluorescence images of WT and *TSC2^−/−^* cells. Inset showing punctate PMEL and fibril ACTA2 staining. Scale bars of 100. B) VEGF‐D secreted into conditioned media measured by ELISA, following 16 h incubation in serum‐free media ± 20 × 10^−9^ m rapamycin (mean ± SD; * = *p* < 0.05 by one‐way analysis of variance (ANOVA); *n* = 9–10). C–E) Visualization of LAM cell invasion after 3 days in hydrogel culture, as C) a schematic, D) brightfield image of single *Z* plane, scale bars of 250 µm, and E) computational reconstruction of cellular spatial positions. F) Median invasion distance of cellular populations plated on the hydrogel and cultured for 3 days ± 20 × 10^−9^ m rapamycin (mean ± SD; * = *p* < 0.05 by one‐way ANOVA with Bonferroni post hoc comparisons; *n* = 124). G) Percentage of cells invaded past fixed threshold set by median invasion distance of genotype‐matched vehicle control. Cells were cultured and treated for 3 days (10 × 10^−6^ m GM6001, a pan‐MMP inhibitor, and 20 × 10^−6^ m Y27632, a ROCK inhibitor, mean ± SD; * = *p* < 0.05 by one‐way ANOVA with Bonferroni post hoc comparisons; *n* = 4). H) Schematic of the sample conditions tested in the bulk RNA‐seq experiment. NT = no treatment, Rapa = rapamycin treatment (20 × 10^−9^ m, 72 h). I) Principal components analysis (PCA) of bulk RNA‐seq samples. J) Heatmap and hierarchal clustering of differentially expressed genes (DEGs) between *TSC2^−/^* ^−^ and WT samples, and between hydrogel and plastic samples, while controlling for the reciprocal covariate. Left panel: transcript expression for plastic and hydrogel cultures was averaged. Right panel: transcript expression for WT and *TSC2^−/−^* samples was averaged. DEG analysis was performed with no treatment samples; genes noted as differentially expressed if FDR < 0.05 and |log_2_FC| > 1. K) Overlap in DEG between genotype and ECM gene lists and LAM cell signature gene list.^[^ ^23^ ^]^ Genes noted as DE if FDR < 0.05. L) Percentage of EdU^+^ (proliferating) cells from 3 h pulse (5 × 10^−6^ m), after 3 days cultured on plastic or hydrogel ± 20 × 10^−9^ m rapamycin (mean ± SD; * = *p* < 0.05 by one‐way ANOVA with Bonferroni post hoc comparisons; *n* = 5).

As pulmonary LAM cells are not amenable to expansion upon lesion explant,^[^ ^13^ ^]^ we established primary cell lines by in vivo differentiation of human pluripotent stem cells (hPSCs), as previously described.^[^ ^21^ ^]^ Briefly, hPSCs were injected into NOD.Cg‐*Prkdc^scid^ Il2rg^tm1Wjl^*/SzJ (NSG) immunodeficient mice to form teratomas, which were explanted and expanded in smooth muscle‐cell enriching conditions (Figure S1A, Supporting Information). We used a previously reported isogenic pair of mCherry^+^ WT and genome‐engineered *TSC2* ^−/−^ generated from the female H9 hPSC cell background of a previously reported allelic series.^[^ ^14^, ^22^ ^]^ Cell cultures exhibit a predominately spindle cell morphology and express *α*‐smooth muscle actin (ACTA2) protein in all isolated cells (**Figure** **1**A and Figure S1B, Supporting Information). Furthermore, immunofluorescence analysis identified a small fraction of PMEL^+^ cells (≈0.13%), a hallmark marker of pulmonary LAM (Figure 1A and Figure S1C, Supporting Information). The high fraction of ACTA2^+^ and low fraction of PMEL^+^ cells in culture is consistent with the relative abundance of these markers in heterogenous human LAM lesions.^[^ ^3^ ^]^ Notably, the percentage of PMEL^+^ and ACTA2^+^ cells did not vary between WT and *TSC2^−/−^* (Figure S1B,C, Supporting Information). Secreted VEGF‐D, a critical biochemical biomarker used in the diagnosis of LAM, was detected in the supernatant of both WT and *TSC2^−/−^* cultures and was insensitive to acute rapamycin treatment (Figure 1B). Together, these data suggest the cell models employed exhibit features of LAM as a product of the cell type isolated, independent of genotype. With this model system, we study the consequence of *TSC2* deficiency specific to a cell context that reflects the human LAM phenotype.

### 2.2 3D Hydrogel Culture Enables Study of the LAM Invasive Phenotype at Single Cell Resolution

We next sought to model the pulmonary invasive phenotype of LAM cells by adapting a lung‐mimetic hydrogel culture system.^[^ ^18^ ^]^ The hydrogel is synthesized by crosslinking hyaluronic acid strands with matrix metalloprotease (MMP)‐cleavable peptides, while embedding vitronectin peptides and methylcellulose to increase cell adhesion and matrix plasticity, respectively. Cells are plated on top of the synthesized hydrogel and actively invade through the material (Figure 1C,D and Movies S1 and S2, Supporting Information). By staining with a nuclear dye and acquiring multiplanar images through the optically clear hydrogel, we identify every cell in *XYZ* planes and compute invasion distances at single cell resolution (Figure S1D, Supporting Information).

We observed all cells from both WT and *TSC2^−/−^* cultures to invade through the hydrogel, albeit at variable distances (Figure 1E). Similar to other LAM characteristics, this suggests that invasion capacity is a product of the cell background isolated and not the genotype. On average, WT cultures invaded further than *TSC2^−/−^* cells in a manner insensitive to acute rapamycin treatment (Figure 1F). We posited that differing invasion distances of cells in the same culture reflect a cell autonomous property, rather than a reflection of stochasticity. To test this, we isolated and expanded clones from WT and *TSC2^−/−^* bulk cultures and subjected these clones to hydrogel culture. We observed a subset of clones with dramatically high invasion speeds, and likewise, a subset with slow invasion speeds (Figure S1E, Supporting Information). These data suggest differential cell autonomous capacities for invasion in putative heterogenous cultures. Finally, we investigated modes of invasion employed by LAM cell models in this hydrogel system. Similar to previous findings,^[^ ^18^ ^]^ we observed a decrease in invasion upon treatment with the pan‐MMP inhibitor GM6001 or the Rho‐kinase (ROCK) inhibitor Y27632, indicating both protease‐dependent and independent modes of invasion employed (Figure 1G).

### 2.3 Loss of *TSC2* and Hydrogel Culture both Confer Transcriptomic Features of LAM

To profile our cell culture system more comprehensively, we conducted bulk RNA‐seq of WT and *TSC2^−/−^* cells, in the presence or absence of rapamycin, and in both plastic and hydrogel culture, for a total of eight sample conditions (Figure 1H). Principal components analysis (PCA) revealed sample genotype to be driving the primary axis of variation, and culture substrate to be driving the secondary axis of variation (Figure 1I). Rapamycin treatment conferred a substantial global transcriptomic change in the *TSC2^−/−^* cells, inducing a profile more similar to WT cells (Figure 1I).

We conducted differential expression analysis comparing across genotype (*TSC2^−/−^* vs WT) and culture substrate (hydrogel vs plastic) in untreated samples, while holding the reciprocal covariate constant. At a false discovery rate (FDR) < 0.05, we identified 6317 differentially expressed genes (DEGs, 1793 with |log_2_FC| > 1) between WT and *TSC2^−/−^*, and 4432 DEGs (771 with |log_2_FC| > 1) between plastic and hydrogel (Table S1A,B and Figure S1F,G, Supporting Information). While exhibiting some overlap, these DEG lists were largely distinct (Figure S1H, Supporting Information). We found 78.8% of the DEGs distinguishing genotype to be reversed by rapamycin treatment, suggesting mTORC1‐dependency (Figure 1J, left panel). In contrast, the expression of DEGs distinguishing plastic versus hydrogel cultures remained largely unchanged in the presence of rapamycin (Figure 1J, right panel). We next examined the overlap of these DEGs with a recently published LAM gene signature derived from single cell RNA‐seq profiling of primary lesions.^[^ ^23^ ^]^ We observed that both DEG lists overlap substantially (65.8% of the total 760 LAM genes) and share both common and distinct genes with the LAM gene signature (Figure 1K).

To glean further biological insight, we conducted Gene Ontology (GO) term enrichment (Table S2A,B and Figure S1I,J, Supporting Information). Both DEG lists ranked “extracellular matrix organization” as most highly enriched, which is also the top enriched term in a primary LAM lesion gene signature list.^[^ ^23^ ^]^ The DEGs distinguishing genotype were also enriched in many terms related to development, similar to primary LAM lesions.^[^ ^23^ ^]^ Interestingly, the DEGs distinguishing culture substrates were largely enriched in GO terms related to proliferation (Table S1B and Figure S1J, Supporting Information). LAM is an indolent disease which progresses at a slow pace relative to other invasive diseases; only a small fraction of cells actively proliferative in primary LAM lesions.^[^ ^3^ ^]^ On plastic, we found WT cells proliferated rapidly, with ≈30% of cells incorporating EdU after a short 3 h pulse (Figure 1L). In contrast, *TSC2^−/−^* cells proliferated at a slightly slower pace, consistent with previous studies of loss of *TSC2* in primary cells.^[^ ^24^ ^]^ Acute rapamycin treatment reduced proliferation of *TSC2^−/−^* cells but did not have a detectable effect on WT cultures. However, subjecting cells to hydrogel culture caused a dramatic decrease in cell proliferation (Figure 1L), likely reflective of the proliferation‐invasion dichotomy.^[^ ^25^ ^]^ Together, these data suggest both genotype (loss of *TSC2*) and culture substrate (3D hydrogel) induce transcriptomic landscapes which model LAM features.

### 2.4 Hydrogel Culture Potentiates Differential mTORC1‐Signaling between WT and *TSC2^−/−^* Cells

**Figure 2:**
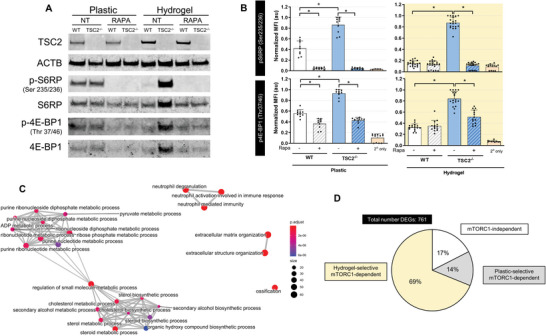
Hydrogel culture potentiates differential mTORC1‐signaling between WT and *TSC2* ^*−/−*^ cells. A) Low input Western blot of protein collected from cells cultured for 3 days on plastic or hydrogel ± 20 × 10^−9^ m rapamycin. NT = no treatment, Rapa = rapamycin treatment. B) Quantification of immunofluorescence values reported in normalized (scaled by replicate maximum value) mean fluorescence intensity. Each point indicates the mean fluorescence intensity from a well of cells cultured on hydrogel or plastic for 3 days ± 20 × 10^−9^ m rapamycin. Secondaryonly values are determined from wells probed with fluorescent secondary antibody only (mean ± SD; * = *p* < 0.05 by one‐way ANOVA with Bonferroni post hoc comparisons; *n* = 9–20). C) Network analysis of GO terms enriched in the list of DEGs found significant (FDR < 0.05) in the interaction between genotype and culture substrate. The 25 most significantly enriched terms are plotted. D) Classification of DEGs according to pattern of expression across genotypes, ECM condition, and in the presence or absence of rapamycin. Gene clusters and classification scheme shown in Figure S2B,C in the Supporting Information.

mTORC1 hyperactivation is a hallmark feature of primary LAM lesions compared to normal adjacent WT tissue. To assess mTORC1 signaling status, we performed a low input western blot, probing for downstream mTORC1 effectors pS6RP and p4E‐BP1. Culturing cells on plastic (2D) showed marginal differences in mTORC1 signaling between WT and *TSC2^−/−^* cells, and both cell types demonstrated activation of mTORC1 above rapamycin‐treated levels (**Figure** **2**A). Remarkably, hydrogel culture potentiated a dramatic difference in mTORC1 signaling, with WT cells downregulating activity to rapamycin‐treated levels and *TSC2^−/−^* cells upregulating signaling above levels seen on plastic alone. This is consistent with the PCA of transcriptomic landscapes, whereby WT untreated and WT rapamycin‐treated samples from hydrogel culture cluster slightly closer together compared to plastic culture (Figure 1I). To corroborate these findings at the single cell level, we examined mTORC1 signaling by immunofluorescence (Figure 2B and Figure S2A, Supporting Information). While a small difference in mTORC1 signaling was observed between WT and *TSC2^−/−^* cells cultured on plastic, this difference was potentiated in 3D hydrogel culture. Importantly, mTORC1 signaling in WT cells was seen to mirror rapamycin‐treated levels only when cultured on hydrogel.

We sought to further explore the genotype‐selective changes induced by hydrogel culture by interrogation of our bulk RNA‐seq dataset. To do so, we tested for genes with a significant coefficient fit to the genotype:substrate interaction term (see Supporting Information and the Experimental Section) and identified 761 DEGs at FDR < 0.05 (Table S1C, Supporting Information). Network analysis of GO terms enriched in this DEG list revealed two principal nodes, one related to sterol synthesis and the other to ribonucleotide metabolism (Figure 2C and Table S2C, Supporting Information). Notably, both these metabolic pathways have been associated with mTORC1 activity.^[^ ^26^ ^]^

To unearth mTORC1‐dependent transcriptomic alterations between WT and *TSC2^−/−^* that differ between plastic and hydrogel culture, we clustered the 761 DEGs based on their expression pattern across the eight experimental conditions (Figure S2B, Supporting Information). Strikingly, genes related to sterol synthesis and ribonucleotide metabolism partitioned largely into two distinct clusters (Figure S2B, Supporting Information). We next classified each gene cluster into one of three categories based on the magnitude of expression differences between WT and *TSC2^−/−^*, and whether the expression changes were rescued by rapamycin (Figure S2C, Supporting Information). Remarkably, we find that 69% of the 761 DEGs showed a greater (or a unique) difference between WT and *TSC2^−/−^* cells in hydrogel culture compared to plastic, which was rescued by rapamycin (Figure 2D). Together, these results demonstrate that hydrogel culture potentiates differential mTORC1 signaling between WT and *TSC2^−/−^* cells, reinforcing a physiologically relevant environment in which mTORC1‐dependent phenotypes can be identified.

### 2.5 3D Drug Screen Identifies Compounds that Modulate Invasion and Cell Viability

**Figure 3:**
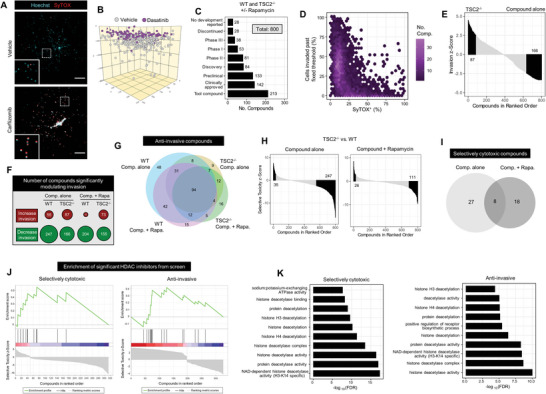
3D drug screen identifies HDAC inhibitors as anti‐invasive and selectively cytotoxic toward *TSC2* ^*−/−*^ LAM cells. A) Representative maximum intensity projection image of *TSC2^−/−^* cells in hydrogel culture for 3 days ± 200 × 10^−9^ m carfilzomib. Scale bars of 250 µm. B) Computational reconstruction of cellular spatial positions following 3 day hydrogel culture of *TSC2^−/−^* cells ± 40 × 10^−9^ m dasatinib. Note that treated and untreated were in separate wells; cells were plotted in the same volume for ease of visualizing relative distances traveled. C) Highest development status reported for the 800 compounds contained in the curated kinase inhibitor and tool compound libraries. A 3D drug screen was conducted on WT and *TSC2^−/−^* cells following 3 day treatment with 5 × 10^−6^ m compounds ± 20 × 10^−9^ m rapamycin. D) Compound invasion modulation plotted against cytotoxicity, aggregating results across genotype and rapamycin treatment. Fixed invasion threshold determined by median invasion distance of untreated controls. Hexagonal plot employed to demonstrate compound densities. E) Waterfall plot of compound invasion *z*‐scores in ranked order; positive values indicate invasion potentiation, while negative values indicate invasion attenuation. Compounds conferring statistically significant invasion modulation highlighted in black. Data presented for *TSC2^−/−^*, no rapamycin treatment condition. F) Number of compounds significantly modulating invasion (potentiating or attenuating) for each genotype in the presence of absence of 20 × 10^−9^ m rapamycin. Bubble area proportional to number of statistically significant targets. G) Overlap of compounds identified as anti‐invasive in each listed condition. H) Waterfall plots of compound selective toxicity *z*‐scores in ranked order; positive values indicate increased cytotoxicity toward *TSC2^−/−^* cells, negative values indicate increased cytotoxicity toward WT cells. Compounds conferring statistically significant selective cytotoxicity highlighted in black. I) Overlap of compounds identified to be selectivity cytotoxic toward *TSC2^−/−^* cells, with or without 20 × 10^−9^ m rapamycin. J) Enrichment plot for compounds annotated to target HDACs, derived from an adapted implementation of GSEA. Hits (black vertical lines) in the red region indicate compounds with a favorable effect, hits in the blue region indicate compounds with an undesirable effect. K) Top 10 most statistically significant GO terms. Analysis performed using targets identified as statistically significantly enriched in screen data by Elion algorithm.

We next employed our hydrogel culture system to identify potential therapeutic compounds. Cell death was measured at the single cell level by application of the live cell imaging fluorophore SyTOX, which selectively permeates cells with compromised plasma membrane integrity. We first tested a known cytotoxic compound, the proteasome inhibitor carfilzomib, and identified substantial cell death by live cell imaging (**Figure** **3**A and Figure S3A, Supporting Information). Additionally, we confirmed the ability to detect invasion modulation effects at the single cell level by employing the known anti‐invasion Src kinase inhibitor dasatinib (Figure 3B and Figure S3B, Supporting Information). To achieve the throughput necessary for a therapeutic screen, we acquired live cell images by high content microscopy paired with automated image analysis tools developed in house. We calculated the drug screen Z’ (a metric for assay quality) to be 0.873 for cytotoxicity measurements and 0.533 for invasion modulation. We subsequently screened a curated library of 800 structurally diverse, bioactive, membrane‐penetrant compounds (Figure 3C and Table S3A, Supporting Information). Of these compounds, 39% have been trialed and shown to be safe for use in humans. We tested one technical replicate of each compound on both WT and *TSC2^−/−^* cells in the presence and absence of rapamycin to elucidate mTORC1‐dependency.

We found a wide variety of compounds with invasion modulatory and cytotoxic capabilities (Table S3B,C, Supporting Information). Unsurprisingly, highly cytotoxic compounds also led to a reduction in bulk invasion (Figure 3D). This trend was independent of genotype and rapamycin treatment (Figure S3C, Supporting Information). However, we observed many compounds which conferred an anti‐invasive effect in the absence of detectable cytotoxicity (Figure 3D and Figure S3C, Supporting Information). We next computed therapeutic invasion *z*‐scores (i.e., statistical measure of compound effect size) by comparing against the vehicle control invasion distribution. In general, more compounds in this library were identified to significantly attenuate rather than potentiate invasion (Figure 3E,F). However, a subset of compounds significantly increased invasion (Figure 3F), a phenotype that would be otherwise overlooked if screening on 2D plastic and could lead to severe adverse consequences in the clinical setting. Importantly, we observed a substantial overlap in the compounds identified to be anti‐invasive across genotypes and treatment conditions, with very few drugs demonstrating a genotype‐selective block to invasion (Figure 3G and Figure S3D, Supporting Information). Together, these data demonstrate the identification of a collection of compounds which block invasion in these cell populations, irrespective of *TSC2* genotype.

A key goal in the therapeutic development landscape for LAM is the identification of compounds which exert selective cytotoxicity toward *TSC2^−/−^* cells. Interestingly, we observed that *TSC2^−/−^* cells exhibited pan‐compound resistance, with over sevenfold more compounds demonstrating significant cytotoxicity toward WT compared to *TSC2^−/−^* cells (Figure 3H). This selectivity is reduced to half with the addition of rapamycin, suggesting generalized resistance is largely due to mTORC1 hyperactivation in *TSC2^−/−^* cells (Figure 3H). We compared the list of compounds that are selectively cytotoxic toward *TSC2^−/−^* cells in the presence versus absence of rapamycin, and observed only a 15% overlap, indicating therapeutic vulnerabilities vary depending on mTORC1 signaling activity (Figure 3I). In summary, we identified a suite of anti‐invasive and selectively cytotoxic therapeutics which can be mined for further development in LAM (Table S3B,C, Supporting Information).

### 2.6 Enrichment Analysis Predicts HDAC Inhibitors as Anti‐Invasive and Selectively Cytotoxic toward *TSC2* ^−/−^ Cells

As our initial drug screen was performed with only one technical replicate, individual compound data are more likely to represent false positives or negatives in comparison to a screen with multiple technical replicates. Thus, to refine our small molecule list for further investigation, we sought to identify groups of outperforming compounds which modulate targets of the same class. Using the known annotated targets of the employed compounds, we performed target enrichment analysis by adapting the Gene Set Enrichment Analysis (GSEA) algorithm. We identified targets conferring well‐established selective cytotoxicity toward *TSC2^−/−^* and anti‐invasive classes, including proteasome inhibition (cytotoxicity) and Src and Rho kinase inhibition (anti‐invasive) (Figure S3E,F and Table S4A–D, Supporting Information). Of note, Src inhibition, a therapeutic route explored in LAM, was found to be selectively cytotoxic toward WT cells (Figure S3E, Supporting Information). Remarkably, pan‐HDAC inhibition was observed to be the only class in the top ten most significant annotations for selective cytotoxicity toward *TSC2^−/−^* and generalized anti‐invasion. We note a substantial favorable enrichment of HDAC‐targeting compounds by both metrics, however, not all compounds annotated to inhibit HDACs performed favorably (Figure 3J).

A limiting factor to our analyses was the small number of compounds which were identified to selectively eliminate *TSC2^−/−^* cells. We sought to extend our compound list in silico using a structure‐based approach with a mechanism of action prediction algorithm (termed Elion). In brief, chemical features are extracted from compound structures and matched with screen performance values to train a machine learning algorithm for prediction of other possibly efficacious compounds. Compounds predicted to be efficacious in silico are then analyzed by target enrichment and pathway analysis. Using this approach, we corroborated HDACs as highly enriched targets for both selective cytotoxicity and anti‐invasion (Table S5A,B, Supporting Information). GO term analysis on significant targets identified nearly all top predicted pathways relate to deacetylation activity, for both selective cytotoxicity and anti‐invasion (Figure 3K and Table S5C,D, Supporting Information). These data together highlighted HDAC inhibitors as promising therapeutics which we explored further and present herein.

### 2.7 HDAC Inhibitors are Selectively Cytotoxic toward *TSC2^−/−^* Cells Exclusively in Hydrogel Culture

**Figure 4:**
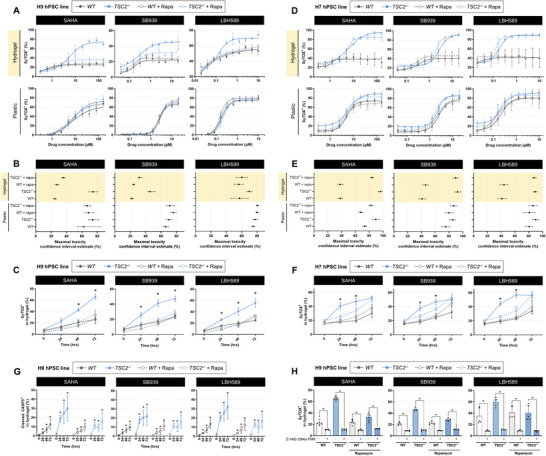
Three safe‐in‐human HDAC inhibitors induce mTORC1‐dependent selective cytotoxicity exclusively in hydrogel culture. A,D) Dose–response cytotoxicity curves of H9 (panel A) and H7 (panel D) cells treated with the indicated HDAC inhibitor for 3 days while cultured on plastic or hydrogel ± 20 × 10^−9^ m rapamycin. Data fit via four‐parameter logistic regression (mean ± SD; *n* = 3). B,E) Confidence intervals of HDAC inhibitor maximal toxicity, estimated by four‐parameter logistic regression models generated in panels (A) and (D). C,F) Percentage of SyTOX^+^ cells following temporal HDAC inhibitor treatment (20 × 10^−6^ m SAHA, 5 × 10^−6^ m SB939, 1 × 10^−6^ m LBH589) of H9 (panel C) and H7 (panel F) cells cultured in hydrogel ± 20 × 10^−9^ m rapamycin (mean ± SD; * = *p* < 0.05 by two‐factor ANOVA with Bonferroni post hoc comparisons; *n* = 3). G) Percentage of cells expressing cleaved caspase 3 (CASP3), following 3 day treatment with HDAC inhibitors in hydrogel (20 × 10^−6^ m SAHA, 5 × 10^−6^ m SB939, and 1 × 10^−6^ m LHB589, mean ± SD; * = *p* < 0.05 by two‐way ANOVA with Dunnett post hoc comparisons to 0 h for each group; *n* = 5–6). H) Percentage of SyTOX^+^ cells following 3 day HDAC inhibitor treatment (20 × 10^−6^ m SAHA, 5 × 10^−6^ m SB939, and 1 × 10^−6^ m LHB589) in hydrogel ± 25 × 10^−6^ m Z‐VAD (OMe)‐FMK (mean ± SD; * = *p* < 0.05 by one‐way ANOVA with Bonferroni post hoc comparisons; *n* = 4–6).

We further tested 11 HDAC inhibitors from our compound library at a wider range of concentrations and identified three to be selectively cytotoxic toward *TSC2^−/−^* cells across a range of concentrations: SAHA (clinically, Vorinostat), SB939 (Pracinostat), and LBH589 (Panobinostat), all of which are pan‐HDAC inhibitors (**Figure** **4**A). We note the atypical therapeutic dose–response curves and selectivity, demonstrating marginal differences in IC_50_ per se but substantial variation in maximal toxicity (Figure 4B). Selective cytotoxicity was largely reversed by co‐treatment with rapamycin, suggesting mTORC1‐dependency. Importantly, the magnitude of cytotoxic selectivity between WT and *TSC2^−/−^* cells increased with treatment duration (Figure 4C). Remarkably, when these HDAC inhibitors were tested with cells cultured on plastic, we did not observe any genotype‐selectivity in their cytotoxic profile (Figure 4A,B). In addition, inhibitor profiles employed in plastic culture did not change in the presence of rapamycin, suggesting a loss of mTORC1‐dependency for cytotoxic effects (Figure 4A,B). We corroborated selective cell death functionally via clonogenic assays (Figure S4A, Supporting Information). While HDAC inhibitors did modulate the proliferation of cells in hydrogel culture, a substantial proliferation blockade was exerted when cells were cultured on plastic, in both genotypes (Figure S4B, Supporting Information).

To appraise patient variability in response to treatment, we next tested these HDAC inhibitors in a second cell background. We generated an isogenic pair of teratoma‐derived smooth muscles cells in the female H7 cell background of a previously reported hPSC allelic series.^[^ ^14^, ^22^ ^]^ Consistent with findings in the H9 cell background, the H7 isogenic pair demonstrated *TSC2^−/−^*‐selective cytotoxicity in response to HDAC inhibitor treatment (Figure 4D,E). This effect was responsive to rapamycin at only 24 and 48 h of treatment duration, suggesting a partial mTORC1‐dependency (Figure 4F). Similar to H9 cells, the genotype‐selective effects were largely abrogated when H7 cells were cultured on plastic, barring SAHA, where a slight increase in toxicity was observed due to the higher baseline *TSC2^−/−^* cell death (Figure 4D,E).

Together, these data indicate a striking difference in cellular responses to HDAC inhibitor treatment while cultured on plastic versus hydrogel. Importantly, HDAC inhibitors only demonstrate mTORC1‐dependent selective toxicity toward *TSC2^−/−^* cells while treated in hydrogel culture. These data are consistent with the observation that hydrogel culture potentiates differential mTORC1 signaling between WT and *TSC2^−/−^* cells (Figure 2 and Figure S2, Supporting Information).

### 2.8 HDAC Inhibitors Induce Cell Death via Apoptosis

We next sought to probe the mode of cell death induced by HDAC inhibitors. Previous studies have provided evidence for both HDAC inhibitor‐mediated apoptosis as well as autophagic cell death.^[^ ^27^ ^]^ Considering we observed a reduction in cell death when co‐treated with rapamycin, a potent inducer of autophagy, we hypothesized the predominant cell death mode to be apoptosis. To test this postulation, we assessed live cell expression of cleaved caspase 3 (CASP3). We validated reagent activity in our hydrogel culture using staurosporine, a known inducer of apoptosis (Figure S4C,D, Supporting Information). For all three HDAC inhibitors, we observed temporal accumulation of cleaved CASP3 with treatment duration in the hydrogel, across all conditions (Figure 4G and Figure S4E, Supporting Information). Importantly, we discerned a complete rescue of cell death by co‐treatment with the caspase inhibitor Z‐VAD (OMe)‐FMK (Figure 4H and Figure S4F,G, Supporting Information). Together, these data demonstrate the employed HDAC inhibitors induce apoptotic cell death in hydrogel culture.

### 2.9 HDAC Inhibitors are Anti‐Invasive, Independent of Cytotoxic Effects

**Figure 5:**
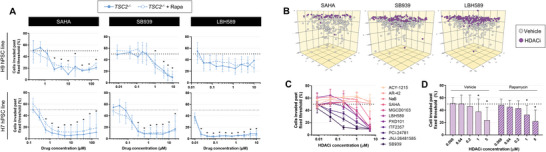
HDAC inhibitors attenuate cell invasion independent of cytotoxicity. A) Live *TSC2^−/−^* cells (H9 top panels, H7 bottom panels) invaded past fixed threshold set by median invasion distance of vehicle control, upon 3 day HDAC inhibitor treatment ± 20 × 10^−9^ m rapamycin (mean ± SD; * = *p* < 0.05 by two‐way ANOVA with Dunnett post hoc comparison to untreated; *n* = 3). B) Computational reconstruction of live cell spatial positions upon 3 day hydrogel culture of *TSC2^−/−^* cells ± HDAC inhibitor (HDACi) treatment (20 × 10^−6^ m SAHA, 5 × 10^−6^ m SB939, 1 × 10^−6^ m LBH589). Note that treated and untreated cells were in separate wells; cells were plotted in the same volume for ease of visualizing relative distances traveled. C) Effect of 11 HDAC inhibitors on *TSC2^−/−^* live cell invasion ± 20 × 10^−9^ m rapamycin. Fixed threshold set by median invasion distance of vehicle control. D) Aggregated effect of the 11 HDAC inhibitors presented in panel (C) (mean ± SD; * = *p* < 0.05 by two‐way ANOVA with Dunnett post hoc comparison to untreated for each group; *n* = 33 via 11 HDACi, *n* = 3 each).

To separate the anti‐invasive effects from cytotoxic effects of these HDAC inhibitors, we identified and computationally removed SyTOX^+^ cells from invasion calculations (Figure S5A, Supporting Information). We determined all three HDAC inhibitors exhibited a dose‐dependent anti‐invasion effect on SyTOX^−^ cells, in both the H9 and H7 cell backgrounds (**Figure** **5**A,B). HDAC inhibitors exerted anti‐invasive effects on both WT and *TSC2^−/−^* cells in the presence or absence of rapamycin, with an effect size that was generally larger in the *TSC2^−/−^* cells (Figure 5A,B and Figure S5B,C, Supporting Information). Remarkably, of the 11 HDAC inhibitors we tested, eight demonstrated anti‐invasive effects in a dose‐dependent manner (Figure 5C). When aggregated as a class of therapeutics, there is a significant increase in anti‐invasive effects with escalating doses, independent of cytotoxicity (Figure 5D). Together, these data demonstrate HDAC inhibitors are effective anti‐invasive agents independent of their cytotoxic profile.

### 2.10 Xenotransplantation of LAM Cell Models into Zebrafish Larvae Permits Dynamic Tracking of Cell Invasion

**Figure 6:**
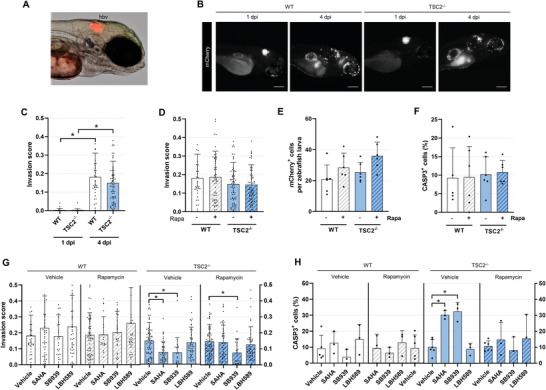
HDAC inhibitors are anti‐invasive and selectively cytotoxic toward TSC2^−/−^ cells xenotransplanted into zebrafish. A) Representative phase contrast image of 1 day post‐injection (dpi) zebrafish larvae injected with *TSC2^−/−^* mCherry^+^ cells into the hindbrain ventricle (hbv). B) Representative images of zebrafish larvae injected with mCherry^+^ WT or *TSC2^−/−^* cells into the hbv. Fish were imaged 1 and 4 dpi. Scale bars of 200 µm. C,D) Quantification of cell invasion using automated invasion analysis. Images analyzed in (D) were taken 4 dpi following 3 day treatment ± 20 × 10^−9^ m rapamycin (mean ± SD; * = *p* < 0.05 by the Kruskal–Wallis test with Dunn's post hoc comparisons; *n* = 38–73). E) Number of mCherry^+^ cells detected per zebrafish following whole larvae dissociation at 4 dpi and analysis by flow cytometry. Samples were treated for 3 days ± 20 × 10^−9^ m rapamycin. Each replicate is a pool of 15–20 zebrafish larvae (mean ± SD; * = *p* < 0.05 by one‐way ANOVA; *n* = 6). F) Percentage of CASP3^+^ cells in the mCherry^+^ population from whole larvae dissociation at 4 dpi, following 3 day treatment ± 20 × 10^−9^ m rapamycin. Each replicate is a pool of 15–20 zebrafish larvae (mean ± SD; * = *p* < 0.05 by one‐way ANOVA; *n* = 5–6). G,H) Effect of 3 day HDAC inhibitor treatment (20 × 10^−6^ m SAHA, 5 × 10^−6^ m SB939, 1 × 10^−6^ m LBH589) ± 20 × 10^−9^ m rapamycin. G) Invasion scores calculated on images acquired 4 dpi (mean ± SD; * = *p* < 0.05 by the Kruskal–Wallis test with Dunn's post hoc comparison to vehicle‐treated; *n* = 27–73). H) Percentage of CASP3^+^ cells in the mCherry^+^ population from whole larvae dissociation at 4 dpi. Each replicate is a pool of 15–20 zebrafish larvae (mean ± SD; * = *p* < 0.05 by ANOVA with Dunnett post hoc comparison to vehicle‐treated; *n* = 3–6). Not all outliers in (C,D) and (G) are visualized due to trimmed axes (although outliers were included in mean ± SD and the statistical calculation).

We next sought to evaluate the in vivo efficacy of the HDAC inhibitors SAHA, SB939, and LBH589. Consistent with previous findings, we found that loss of *TSC2* alone was insufficient to confer tumorigenicity upon subcutaneous xenotransplantation in immunodeficient mice (Figure S6A, Supporting Information). To avoid immortalization of our cell models—a process which dramatically alters cellular characteristics—we performed a well‐established xenotransplantation assay in zebrafish larvae.^[^ ^28^, ^29^ ^]^ In this system, WT or *TSC2^−/−^* cells are injected into the hindbrain ventricle of zebrafish larvae 3 days post‐fertilization, imaged 1 day post‐injection (dpi) to ensure successful engraftment, and then imaged again at 4 dpi to visualize local invasion (**Figure** **6**A,B). Cells were tracked by their endogenous mCherry expression.^[^ ^14^ ^]^ The optical clarity of this system provides the advantage of enabling isogenic comparisons between WT and *TSC2^−/−^* human cells in vivo while dynamically tracking cell invasion.

To quantify invasion in an unbiased fashion, we computed the ratio of mCherry signal found outside the injection site compared to within (Figure S6B,C, Supporting Information). Using this method, we accurately detect near zero invasion scores 1 dpi, followed by a substantial increase 4 dpi (Figure 6C). We observed comparable invasion scores between WT and *TSC2^−/−^* cells which were unaffected by rapamycin treatment, consistent with in vitro data (Figure 6D). To quantify human cell proliferation and cell death, we digested and pooled whole larvae (15–20 per condition) followed by flow cytometry analysis, probing for mCherry and human‐specific CASP3. Consistent with xenotransplantation in mice, these cells were not tumorigenic and the rate of clearance outstripped proliferation (Figure S6D, Supporting Information). The number of cells at 4 dpi was comparable between genotypes and unaffected by rapamycin treatment (Figure 6E). The percentage of CASP3^+^ cells in the mCherry^+^ population was ≈10% and equivalent across conditions, similar to baseline cell death rates seen in hydrogel culture (Figures 3A and 6F).

### 2.11 HDAC Inhibitors SAHA and SB939 Block Cell Invasion and Selectively Eradicate *TSC2^−/−^* Cells In Vivo

We next employed our zebrafish xenograft system to assess the efficacy of HDAC inhibitors in vivo. To achieve the highest quality of preclinical evidence, experiments were conducted in a randomized, double‐blinded, placebo‐controlled fashion. We first established dose‐toxicity profiles for each HDAC inhibitor: SB939 and LBH589 conferred an IC_50_ of 53.1 × 10^−6^ and 6.74 × 10^−6^ m, respectively, while the favorable toxicity profile of SAHA precluded calculation of an IC_50_ value (Figure S6E, Supporting Information). Of note, in vivo HDAC inhibitor potency correlated with the in vitro cytotoxicity profile. Zebrafish engrafted with either WT or *TSC2^−/−^* cells were treated with HDAC inhibitors by immersion therapy, in the presence or absence of rapamycin. Importantly, we used the same compound concentration as those employed in vitro, which was well below each compound's IC_50_ value.

After 3 days of treatment, we observed that SAHA and SB939, but not LBH589, exerted a statistically significant anti‐invasive effect in the absence of rapamycin, exclusively on the *TSC2^−/−^* cells (Figure 6G). SB939 also demonstrated a statistically significant anti‐invasive effect in the presence of rapamycin. We note that live cells could not be distinguished from dead or dying cells in this quantification. However, by flow cytometry, we observed an increase in the percentage of human *TSC2^−/−^* cells to be CASP3^+^ upon treatment with SAHA and SB939, but not LBH589 (Figure 6H). This effect was abrogated upon combination treatment with rapamycin and was not observed in the human WT cells. Together, these data indicate the HDAC inhibitors SAHA and SB939 exhibit in vivo anti‐invasion and selective cytotoxicity effects toward *TSC2^−/−^* cells.

## 3 Conclusion

Here, we employ tissue engineering tools to model LAM and identify new therapeutic strategies. We find that synthetic hydrogel culture of LAM cells more faithfully recapitulates the molecular and phenotypic characteristics of the human disease, in comparison to culture on plastic. We identified HDAC inhibitors as anti‐invasive and selectively cytotoxic toward *TSC2^−/−^* cells, both in vitro and in vivo. Importantly, selective cytotoxicity is seen exclusively in hydrogel culture and abrogated on plastic (Figure 4A,B,D,E). These findings demonstrate tissue‐engineered disease modeling exposes physiologically relevant therapeutic vulnerabilities that would be otherwise missed by conventional culture conditions.

Previous studies have demonstrated that hydrogel culture of cell lines is more predictive of in vivo drug sensitivities compared to conventional culture on 2D plastic.^[^ ^30^, ^31^ ^]^ In the context of monogenic disease such as LAM, preclinical investigations involve comparison of the affected genotype (i.e. *TSC2^−/−^)* against matched WT controls. We show HDAC inhibition confers genotype‐selective cytotoxicity exclusively in hydrogel culture. This therapeutic vulnerability arises from potentiated differential mTORC1 signaling between WT and *TSC2^−/−^* cells compared to culture on plastic (Figure 2 and Figure S2, Supporting Information). It is unknown which mechanochemical property of the hydrogel confers this differential mTORC1 signaling, and multiple variables may be involved, including elasticity, viscosity, ligand availability, polymer architecture, and so forth. Interestingly, focal adhesion kinase (FAK) has been shown to exhibit adhesion‐induced mTORC1 activation via TSC2,^[^ ^32^ ^]^ situating elastic mechanotransduction as one possible mechanism. Nonetheless, hyperactive mTORC1 signaling is a hallmark feature of LAM,^[^ ^7^ ^]^ highlighting the physiologically relevant environment presented by hydrogel culture. Furthermore, the variable rescue effect of rapamycin across cell backgrounds recapitulates the clinically heterogenous response of LAM patients to rapamycin treatment, underscoring the utility in analyzing multiple donor backgrounds, even when performing isogenic comparisons.^[^ ^11^, ^12^ ^]^

The findings presented in this article complement a recent study evidencing therapeutic efficacy of HDAC inhibitors in a *Tsc1^−/−^*‐driven mouse model of lymphangiosarcoma.^[^ ^33^ ^]^ HDAC inhibitors present an opportune class of molecules for pursuit due to the wide variety of compounds already approved for clinical use. Indeed, both SAHA and LBH589 are approved for use in cutaneous T cell lymphoma and multiple myeloma, respectively.^[^ ^34^, ^35^ ^]^ The safe‐in‐human toxicity profile of these compounds will facilitate rapid translation for testing in LAM patients. Importantly, our employed HDAC inhibitors exhibit selective cytotoxicity in an mTORC1‐dependent manner, suggesting generalizable efficacy to mTORC1‐driven malignancies. Of note, cutaneous T cell lymphoma cells have been observed to exhibit mTORC1 hyperactivation compared to matched normal controls.^[^ ^36^ ^]^

In this work, we used equivalent HDAC inhibitor concentrations for in vitro and in vivo experiments; these concentrations were well‐below dose‐limiting toxicities in zebrafish (Figure S6E, Supporting Information). However, a critical outstanding question is whether the concentrations employed are physiologically attainable in humans. Pharmacokinetic studies of SAHA, SB939, and LBH589 in humans have demonstrated micromolar serum concentrations are achievable.^[^ ^37^, ^38^, ^39^ ^]^ In fact, the original preclinical work which formed the foundation for testing SAHA as a treatment in cutaneous T cell lymphoma used the drug in vitro at micromolar concentrations.^[^ ^40^ ^]^ Thus, we anticipate drug concentrations necessary to elicit a therapeutic effect are achievable in patients with LAM.

While HDAC inhibitors largely demonstrated efficacy in the zebrafish xenografts, differences between in vitro and in vivo were observed. LBH589 did not exhibit any detectable anti‐invasive or cytotoxic effects in vivo, which may arise from possible altered bioavailability in the zebrafish. While SAHA and SB939 were both anti‐invasive and cytotoxic toward the *TSC2^−/−^* xenografts (which was rescued by rapamycin treatment), anti‐invasive effects on the WT xenografts were not detected. As we could not partition out viable xenografted cells, this raises the possibility that anti‐invasion was an exclusive consequence of cytotoxicity. However, this postulation is undermined by the observation that SB939 combined with rapamycin exerted an anti‐invasive effect, despite a lack of cytotoxicity. More likely, we hypothesize that the lack of detectable in vivo anti‐invasion effects on the WT cells is a consequence of the increased variability of the zebrafish assay, which reduced our statistical power and thus ability to detect smaller effect sizes.

Throughout our study, we note a variety of LAM features in our cellular models that exist independently from loss of *TSC2*. For example, cells isolated from both WT and *TSC2^−/−^* teratomas are equally invasive, present matching ACTA2^+^/PMEL^+^ profiles, and secrete similar levels of VEGF‐D. Indeed, similar observations of LAM features in WT cells have been noted in a neural crest cell model.^[^ ^14^ ^]^ These data suggest perhaps, while loss of *TSC2* is critical for disease pathogenesis, the hallmark features of the putative “LAM cell” may already exist in a physiological, if not transient, context (e.g., during development, injury repair, and inflammation). Critical consideration of the cell context is essential, even while employing isogenic comparisons, as different cell types exhibit distinct therapeutic vulnerabilities.^[^ ^14^ ^]^

In summary, we have identified HDAC inhibitors as anti‐invasive and selectively cytotoxic toward *TSC2^−/−^* cells in vitro and in vivo. This therapeutic vulnerability of LAM cells was only exposed upon synthetic hydrogel culture and would have been missed if culturing on plastic. On the path toward clinical translation, we anticipate testing of these compounds in more diverse disease models. By validating compounds with physiologically relevant orthogonal tools and techniques, we may elevate the most promising therapeutic for clinical trials.

## 4 Experimental Section

Detailed experimental methods are provided in the Supporting Information.

### Ethics and Animal Husbandry

All animal experiments were conducted with approval from the University of Ottawa Animal Care Committee (Protocols #OHRI1666 and #CHEOe‐3171), in accordance with the Canadian Council on Animal Care Standards and the Province of Ontario's Animals for Research Act. NSG mice (Jackson Laboratory) were maintained in sterile housing conditions and fed autoclaved chow and water ad libitum. Adult *casper* ^[^ ^41^ ^]^ zebrafish (a gift from Dr. Leonard Zon, Boston Children's Hospital, Boston, MA) were maintained in a recirculating commercial housing system (Aquatic Habitats, now Pentair) at 28 °C in 14 h:10 h light:dark conditions in the aquatics facility at the University of Ottawa, Ottawa, ON. Adult *casper* zebrafish were bred according to standard protocol,^[^ ^42^ ^]^ and embryos were collected and grown in E3 medium (5 × 10^−3^ m NaCl, 0.17 × 10^−3^ m KCl, 0.33 × 10^−3^ m CaCl_2_, 0.33 × 10^−3^ m MgSO_4_) at 28 °C in 10 cm Petri dishes until the desired time point. Embryos were cleaned and provided with new media every 24 h.

## Conflict of Interest

The authors declare no conflict of interest.

## Author Contributions

Conceptualization: A.P., J.Y.L., S.P.D., R.Y.T., J.N.B., M.S.S., W.L.S., Methodology: A.P., J.Y.L., N.M., L.J.S., N.A., C.X., N.M., L.M.J., R.Y.T., M.S.S., W.L.S., Investigation: A.P., J.Y.L., N.M., N.A., N.M., E.L., A.C., C.D., Visualization: A.P., J.Y.L., Funding acquisition: M.S.S., W.L.S., Supervision: G.M., L.M.J., A.S.K., R.Y.T., J.N.B., M.S.S., W.L.S., Writing—original draft: A.P., W.L.S., Writing—review & editing: A.P., J.Y.L., N.M., L.J.S., S.P.D., N.A., C.X., E.L., A.C., C.D., G.M., L.M.J., A.S.K., R.Y.T., J.N.B., M.S.S., W.L.S.

## Supporting information

Supporting Information

Supplemental Movie 1

Supplemental Movie 2

Supplemental Table 1

Supplemental Table 2

Supplemental Table 3

Supplemental Table 4

Supplemental Table 5

## Data Availability

The data that support the findings of this study are openly available in GEO at https://www.ncbi.nlm.nih.gov/geo/query/acc.cgi?acc=GSE179044, reference number 179044.

## References

[1] A. M. Taveira‐DaSilva , J. Moss , Clin. Epidemiol. 2015, 7, 249. 25897262 10.2147/CLEP.S50780 PMC4396456

[2] J. Moss , N. A. Avila , P. M. Barnes , R. A. Litzenberger , J. Bechtle , P. G. Brooks , C. J. Hedin , S. Hunsberger , A. S. Kristof , Am J. Respir. Crit. Care Med. 2001, 164, 669. 11520735 10.1164/ajrccm.164.4.2101154

[3] X. Zhe , L. Schuger , J. Histochem. Cytochem. 2004, 52, 1537. 15557209 10.1369/jhc.4A6438.2004

[4] G. F. Abbott , M. L. Rosado‐de‐Christenson , A. A. Frazier , T. J. Franks , R. D. Pugatch , J. R. Galvin , Radiographics 2005, 25, 803. 15888627 10.1148/rg.253055006

[5] S. C. Chu , K. Horiba , J. Usuki , N. A. Avila , C. C. Chen , W. D. Travis , V. J. Ferrans , J. Moss , Chest 1999, 115, 1041. 10208206 10.1378/chest.115.4.1041

[6] T. Urban , R. Lazor , J. Lacronique , M. Murris , S. Labrune , D. Valeyre , J. F. Cordier , Medicine 1999, 78, 321. 10499073 10.1097/00005792-199909000-00004

[7] E. P. Henske , F. X. McCormack , J. Clin. Invest. 2012, 122, 3807. 23114603 10.1172/JCI58709 PMC3484429

[8] A. M. Taveira‐DaSilva , O. Hathaway , M. Stylianou , J. Moss , Ann. Intern. Med. 2011, 154, 797. 21690594 10.1059/0003-4819-154-12-201106210-00007 PMC3176735

[9] J. Bee , S. Fuller , S. Miller , S. R. Johnson , Thorax 2018, 73, 369. 28993539 10.1136/thoraxjnl-2017-210872

[10] J. Yao , A. M. Taveira‐DaSilva , A. M. Jones , P. Julien‐Williams , M. Stylianou , J. Moss , Am. J. Respir. Crit. Care Med. 2014, 190, 1273. 25329516 10.1164/rccm.201405-0918OC PMC4315813

[11] F. X. McCormack , Y. Inoue , J. Moss , L. G. Singer , C. Strange , K. Nakata , A. F. Barker , J. T. Chapman , M. L. Brantly , J. M. Stocks , K. K. Brown , J. P. Lynch , H. J. Goldberg , L. R. Young , B. W. Kinder , G. P. Downey , E. J. Sullivan , T. V. Colby , R. T. McKay , M. M. Cohen , L. Korbee , A. M. Taveira‐DaSilva , H.‐S. Lee , J. P. Krischer , B. C. Trapnell , N. Engl. J. Med. 2011, 364, 1595. 21410393 10.1056/NEJMoa1100391 PMC3118601

[12] J. J. Bissler , F. X. McCormack , L. R. Young , J. M. Elwing , G. Chuck , J. M. Leonard , V. J. Schmithorst , T. Laor , A. S. Brody , J. Bean , S. Salisbury , D. N. Franz , N. Engl. J. Med. 2008, 358, 140. 18184959 10.1056/NEJMoa063564 PMC3398441

[13] E. A. Goncharova , D. A. Goncharov , A. Eszterhas , D. S. Hunter , M. K. Glassberg , R. S. Yeung , C. L. Walker , D. Noonan , D. J. Kwiatkowski , M. M. Chou , R. A. Panettieri , V. P. Krymskaya , J. Biol. Chem. 2002, 277, 30958. 12045200 10.1074/jbc.M202678200

[14] S. P. Delaney , L. M. Julian , A. Pietrobon , J. Yockell‐Lelièvre , C. Doré , T. T. Wang , V. C. Doyon , A. Raymond , D. A. Patten , A. S. Kristof , M.‐E. Harper , H. Sun , W. L. Stanford , bioRxiv 2020, 683359.

[15] D. J. Kwiatkowski , Lymphatic Res. Biol. 2010, 8, 51. 10.1089/lrb.2009.0013 PMC2883495 20235887

[16] S. R. Caliari , J. A. Burdick , Nat. Methods 2016, 13, 405. 27123816 10.1038/nmeth.3839 PMC5800304

[17] J. A. Burdick , G. D. Prestwich , Adv. Mater. 2011, 23, H41. 21394792 10.1002/adma.201003963 PMC3730855

[18] R. Y. Tam , J. Yockell‐Lelièvre , L. J. Smith , L. M. Julian , A. E. G. Baker , C. Choey , M. S. Hasim , J. Dimitroulakos , W. L. Stanford , M. S. Shoichet , Adv. Mater. 2019, 31, 1806214. 10.1002/adma.201806214 30589121

[19] C. A. MacRae , R. T. Peterson , Nat. Rev. Drug Discovery 2015, 14, 721. 26361349 10.1038/nrd4627

[20] D. C. Swinney , J. Anthony , Nat. Rev. Drug Discovery 2011, 10, 507. 21701501 10.1038/nrd3480

[21] L. M. Julian , S. P. Delaney , Y. Wang , A. A. Goldberg , C. Doré , J. Yockell‐Lelièvre , R. Y. Tam , K. Giannikou , F. McMurray , M. S. Shoichet , M.‐E. Harper , E. P. Henske , D. J. Kwiatkowski , T. N. Darling , J. Moss , A. S. Kristof , W. L. Stanford , Cancer Res. 2017, 77, 5491. 28830860 10.1158/0008-5472.CAN-17-0925 PMC5645248

[22] A. Pietrobon , J. Yockell‐Lelièvre , T. A. Flood , W. L. Stanford , Cell Rep. 2022, 40, 111048. 35793620 10.1016/j.celrep.2022.111048

[23] M. Guo , J. J. Yu , A. K. Perl , K. A. Wikenheiser‐Brokamp , M. Riccetti , E. Y. Zhang , P. Sudha , M. Adam , A. Potter , E. J. Kopras , K. Giannikou , S. S. Potter , S. Sherman , S. R. Hammes , D. J. Kwiatkowski , J. A. Whitsett , F. X. McCormack , Y. Xu , Am J. Respir. Crit. Care Med. 2020, 202, 1373. 32603599 10.1164/rccm.201912-2445OC PMC7667901

[24] H. Zhang , G. Cicchetti , H. Onda , H. B. Koon , K. Asrican , N. Bajraszewski , F. Vazquez , C. L. Carpenter , D. J. Kwiatkowski , J. Clin. Invest. 2003, 112, 1223. 14561707 10.1172/JCI17222 PMC213485

[25] A. Giese , M. A. Loo , N. Tran , D. Haskett , S. W. Coons , M. E. Berens , Int. J. Cancer 1996, 67, 275. 8760599 10.1002/(SICI)1097-0215(19960717)67:2<275::AID-IJC20>3.0.CO;2-9

[26] A. J. Valvezan , B. D. Manning , Nat. Metab. 2019, 1, 321. 32694720 10.1038/s42255-019-0038-7 PMC12569966

[27] D. S. Schrump , Clin. Cancer Res. 2009, 15, 3947. 19509170 10.1158/1078-0432.CCR-08-2787 PMC6354580

[28] B. Adane , G. Alexe , B. K. A. Seong , D. Lu , E. E. Hwang , D. Hnisz , C. A. Lareau , L. Ross , S. Lin , F. S. Dela Cruz , M. Richardson , A. S. Weintraub , S. Wang , A. B. Iniguez , N. V. Dharia , A. S. Conway , A. L. Robichaud , B. Tanenbaum , J. M. Krill‐Burger , F. Vazquez , M. Schenone , J. N. Berman , A. L. Kung , S. A. Carr , M. J. Aryee , R. A. Young , B. D. Crompton , K. Stegmaier , Cancer Cell 2021, 39, 827. 34129824 10.1016/j.ccell.2021.05.007 PMC8378827

[29] A. M. El‐Naggar , C. J. Veinotte , H. Cheng , T. G. P. Grunewald , G. L. Negri , S. P. Somasekharan , D. P. Corkery , F. Tirode , J. Mathers , D. Khan , A. H. Kyle , J. H. Baker , N. E. LePard , S. McKinney , S. Hajee , M. Bosiljcic , G. Leprivier , C. E. Tognon , A. I. Minchinton , K. L. Bennewith , O. Delattre , Y. Wang , G. Dellaire , J. N. Berman , P. H. Sorensen , Cancer Cell 2015, 27, 682. 25965573 10.1016/j.ccell.2015.04.003

[30] R. Edmondson , J. J. Broglie , A. F. Adcock , L. Yang , Assay Drug Dev. Technol. 2014, 12, 207. 24831787 10.1089/adt.2014.573 PMC4026212

[31] A. E. G. Baker , L. C. Bahlmann , R. Y. Tam , J. C. Liu , A. N. Ganesh , N. Mitrousis , R. Marcellus , M. Spears , J. M. S. Bartlett , D. W. Cescon , G. D. Bader , M. S. Shoichet , Adv. Mater. 2019, 31, 1901166. 10.1002/adma.201901166 31322299

[32] B. Gan , Y. Yoo , J.‐L. Guan , J. Biol. Chem. 2006, 281, 37321. 17043358 10.1074/jbc.M605241200

[33] F. Yang , S. Sun , C. Wang , M. Haas , S. Yeo , J.‐L. Guan , Br. J. Cancer 2020, 122, 1791. 32336756 10.1038/s41416-020-0839-1 PMC7283252

[34] B. S. Mann , J. R. Johnson , M. H. Cohen , R. Justice , R. Pazdur , Oncologist 2007, 12, 1247. 17962618 10.1634/theoncologist.12-10-1247

[35] L. A. Raedler , Am. Health Drug Benefits 2016, 9, 84. PMC5013857 27668050

[36] T. E. Witzig , C. Reeder , J. J. Han , B. LaPlant , M. Stenson , H. W. Tun , W. Macon , S. M. Ansell , T. M. Habermann , D. J. Inwards , I. N. Micallef , P. B. Johnston , L. F. Porrata , J. P. Colgan , S. Markovic , G. S. Nowakowski , M. Gupta , Blood 2015, 126, 328. 25921059 10.1182/blood-2015-02-629543 PMC4504947

[37] E. H. Rubin , N. G. B. Agrawal , E. J. Friedman , P. Scott , K. E. Mazina , L. Sun , L. Du , J. L. Ricker , S. R. Frankel , K. M. Gottesdiener , J. A. Wagner , M. Iwamoto , Clin. Cancer Res. 2006, 12, 7039. 17145826 10.1158/1078-0432.CCR-06-1802

[38] F. Giles , T. Fischer , J. Cortes , G. Garcia‐Manero , J. Beck , F. Ravandi , E. Masson , P. Rae , G. Laird , S. Sharma , H. Kantarjian , M. Dugan , M. Albitar , K. Bhalla , Clin. Cancer Res. 2006, 12, 4628. 16899611 10.1158/1078-0432.CCR-06-0511

[39] W. P. Yong , B. C. Goh , R. A. Soo , H. C. Toh , K. Ethirajulu , J. Wood , V. Novotny‐Diermayr , S. C. Lee , W. L. Yeo , D. Chan , D. Lim , E. Seah , R. Lim , J. Zhu , Ann. Oncol. 2011, 22, 2516. 21385886 10.1093/annonc/mdq784

[40] C. Zhang , V. Richon , X. Ni , R. Talpur , M. Duvic , J. Invest. Dermatol. 2005, 125, 1045. 16297208 10.1111/j.0022-202X.2005.23925.x

[41] R. M. White , A. Sessa , C. Burke , T. Bowman , J. LeBlanc , C. Ceol , C. Bourque , M. Dovey , W. Goessling , C. E. Burns , L. I. Zon , Cell Stem Cell 2008, 2, 183. 18371439 10.1016/j.stem.2007.11.002 PMC2292119

[42] M. Westerfield , The Zebrafish Book. A Guide for the Laboratory Use of Zebrafish (Danio rerio), University of Oregon Press, Eugene, OR 2000.

