# Supplementary material for: Tissue‐Engineered Disease Modeling of Lymphangioleiomyomatosis Exposes a Therapeutic Vulnerability to HDAC Inhibition
Source: Adv Sci (Weinh). 2023 Jul 3;10(26):2302611. doi: 10.1002/advs.202302611 (PMC10502849; doi:10.1002/advs.202302611)
Supplement: Supplementary file 1 — Supporting Information [file ADVS-10-2302611-s005.pdf]

ADVANCED SCIENCE Open Access

Supporting Information

for *Adv. Sci.*, DOI 10.1002/advs.202302611

Tissue-Engineered Disease Modeling of Lymphangioleiomyomatosis Exposes a Therapeutic Vulnerability to HDAC Inhibition

*Adam Pietrobon, Julien Yockell-Lelièvre, Nicole Melong, Laura J. Smith, Sean P. Delaney, Nadine Azzam, Chang Xue, Nishanth Merwin, Eric Lian, Alberto Camacho-Magallanes, Carole Doré, Gabriel Musso, Lisa M. Julian, Arnold S. Kristof, Roger Y. Tam, Jason N. Berman, Molly S. Shoichet and William L. Stanford**

Supporting Information

Tissue-Engineered Disease Modeling of Lymphangioleiomyomatosis Exposes a Therapeutic Vulnerability to HDAC Inhibition

Adam Pietrobon, Julien Yockell-Lelièvre, Nicole Melong, Laura J. Smith, Sean P. Delaney, Nadine Azzam, Chang Xue, Nishanth Merwin, Eric Lian, Alberto Camacho-Magallanes, Carole Doré, Gabriel Musso, Lisa M. Julian, Arnold S. Kristof, Roger Y. Tam, Jason N. Berman, Molly S. Shoichet, William L. Stanford

Table of contents

Experimental methods **4**

Study design 4

Statistical analysis 5

Study reagents and resources 6

Cell culture 8

Pluripotent stem cell culture 8

E8 media recipe 8

LAM and control cell model derivation and culture 8

LAM and control cell model clonal isolation 9

Hydrogel culture 10

Reagent production 10

Hydrogel gelation and culture 10

Cell treatments 10

Drug treatments 11

Live cell staining 11

Cytotoxicity-invasion assay 11

Microscopy 12

Three-dimensional drug screen 12

Screen design 12

Compound score calculation 13

Target enrichment analysis 13

Elion™ analysis 14

Image analysis 16

Identification of cell spatial positions and cell invasion distance 16

Binarization of live cell stains 17

Quantification of cellular invasion 18

Immunofluorescence stain quantification 18

Invasion quantification upon zebrafish xenotransplantation 19

RNA-seq 19

RNA extraction and quality control 19

RNA-sequencing and raw data processing 20

Differential gene expression and enrichment analysis 20

Animal studies 21

Mouse teratoma formation 21
Mouse subcutaneous xenografts 22
Mouse IVIS image acquisition........ 22
Zebrafish toxicity assay 22
Zebrafish hindbrain ventricle xenotransplantation 22
Zebrafish drug treatments 23
Zebrafish whole larval dissociation and fixation 24
Immunofluorescence staining 24
Enzyme-linked immunosorbent assay (ELISA) 25
Flow cytometry 25
Low input western blot 26
EdU proliferation assay 27
Clonogenic assay 28
Supplementary tables 29
Supplementary movies........ 31
Supplementary figures........ 32
References 41

Experimental methods

Study design

The objective of this research was to assess the LAM disease modelling capabilities of newly developed tissue-engineered cell models, and subsequently employ these models to identify novel therapeutic compounds. We conducted a 3D drug screen, and based on the acquired data, formulated and tested the following hypothesis: HDAC inhibitors are anti-invasive and selectively cytotoxic towards *TSC2*$^{-/-}$ cells. We employed a combination of *in vitro* and *in vivo* tools to test this hypothesis. Drug screen data was analyzed in a blinded, unbiased manner, and independently by two different researchers using distinct methods. Unless otherwise stated, all *in vitro* data presented is from the H9 cell background. Animal studies were conducted and analyzed in a double-blinded, randomized, placebo-controlled manner to generate the highest quality pre-clinical evidence. Blinding was achieved by codification of an investigator uninvolved in the experiments performed. A variety of experimental tools were employed to interrogate this hypothesis, described in the subsequent Supplementary Materials and Methods. All reagents used and concentrations employed (if relevant) are reported in the Supplementary Materials and Methods.

Sample sizes for both *in vitro* and *in vivo* studies were determined according to field-specific conventions. Power analysis was not employed. Data collection was not stopped prematurely, and every experimental replicate was analyzed. All data points were included in the data presentation; outliers were only excluded if there was definitive empirical evidence of technical error and noted as such in the figure legend. Experiments were repeated at least three times unless otherwise noted, with replicates collected at separate points in time and under

independent conditions. RNA-seq data is accessible at the Gene Expression Omnibus (GEO) repository with accession GSE179044.

Statistical analysis

All figures are presented with individual data points (where graphically appropriate), with measures of central tendency and error to be mean and standard deviation, respectively, as stated in each figure legend. Sample sizes (n), statistical testing procedures, and post-hoc analyses employed are also reported in each figure legend, with significance (*) attributed when $p < 0.05$. In general, for normally distributed data sets with equal variances, we performed unpaired two-tailed t-tests, or one-way or two-way ANOVA testing followed by Bonferroni post-hoc testing (for select pairings) or Dunnett post-hoc testing (for repeated comparisons to control groups). For zebrafish invasion data, non-normal distributions were observed, so we compared groups with Kruskal-Wallis testing followed by Dunn's post-hoc testing. Dose-response curves were fit with a four-parameter logistic regression. All figures included data from at least three independent experiments, without removal of statistical outliers.

H9 data were analyzed and reported in an untransformed format. For hydrogel cytotoxicity data generated in the H7 cell background, we observed high inter-replicated variability in basal toxicity. To correct for batch effect, we normalized values to their intra-replicate control condition (e.g. untreated at t = 0h of the same genotype) and then adjusted by the average control condition value. Thus, data across all replicates were processed with the following linear transformation: $S_{rxA} = S_{mxA} + (S_{cT} – S_{cA})$, where S_{rxA} is the % SyTOX^{+} cells reported for treatment x in replicate A, S_{mxA} is the % SyTOX^{+} cells measured for treatment x in replicate A, S_{cT} is the average % SyTOX^{+} cells measured for controls across all replicates, and

S_{cA} is the % SyTOX^{+} cells measured for controls in replicate A. Additional statistical testing procedures, including calculation of drug screen statistics (e.g., z-scores, selectivity scores), are described further in the **Experimental methods** section. All statistical testing was performed using the software GraphPad Prism 9.

Study reagents and resources

Please see below (**Table 1**) for a list of key reagents and resources used in this study.

Table 1: List of key reagents and resources used in this study.

REAGENTS and RESOURCES	SOURCE	IDENTIFIER
Antibodies		
PMEL	Thermo Fisher Scientific	MA5-13232
ACTA2	Abcam	ab5694
mCherry	Abcam	ab167453
pS6RP (Ser 235/236)	CST	2211
p4E-BP1(Thr 37/46)	CST	2855
S6RP	CST	2317
4E-BP1	Thermo Fisher Scientific	AHO1382
TSC2	CST	4308
ACTB	CST	370
Cleaved CASP3	CST	9661
mCherry	Thermo Fisher Scientific	M11217
AlexaFluor 488 Goat Anti-Rabbit IgG (H+L)	Thermo Fisher Scientific	A11034
AlexaFluor 488 Goat Anti-Mouse IgG (H+L)	Thermo Fisher Scientific	A11001
AlexaFluor 555 Goat Anti-Rabbit IgG (H+L)	Thermo Fisher Scientific	A21428
AlexaFluor 555 Goat Anti-Mouse IgG (H+L)	Thermo Fisher Scientific	A32727
AlexaFluor 647 Goat Anti-Rabbit IgG (H+L)	Thermo Fisher Scientific	A21245
AlexaFluor 647 Goat Anti-Mouse IgG (H+L)	Thermo Fisher Scientific	A21235
AlexaFluor 680 Goat Anti-Rabbit IgG (H+L)	Thermo Fisher Scientific	A21076
AlexaFluor 680 Goat Anti-Mouse IgG (H+L)	Thermo Fisher Scientific	A28183
DyLight 800 Goat Anti-Rabbit IgG (H+L)	Thermo Fisher Scientific	SA535571
DyLight 800 Goat Anti-Mouse IgG (H+L)	Thermo Fisher Scientific	SA535521
Live cell imaging dyes		
Hoechst 33342	Thermo Fisher Scientific	H3570
SyTOX Green	Thermo Fisher Scientific	S7020
NucView 488 Caspase-3 Enzyme Substrate	Biotium	10402
Small molecules		
Kinase Inhibitor library	Ontario Institute for Cancer Research	N/A
Tool Compound library	Ontario Institute for Cancer Research	N/A
Rapamycin	MilliporeSigma	553211

Staurosporine	AdooQ Bioscience	A10864
SAHA	AdooQ Bioscience	A10979
SB939	AdooQ Bioscience	A10830
LBH589	AdooQ Bioscience	A10518
BGT226	AdooQ Bioscience	A11162
OSI-027	Toronto Research Chemicals	A611910
AZD8055	AdooQ Bioscience	A10114
Y-27632	AdooQ Bioscience	A11001
PR-171 (Carfilzomib)	AdooQ Bioscience	A11278
BMS-354825 (Dasatinib)	AdooQ Bioscience	A10290
EdU	Thermo Fisher Scientific	A10044
Sulfo-Cy5-N_3	Lumiprobe	A333
Buprenophrine	Provided by animal facility	N/A
Hydrogel reagents		
Sodium hyaluronate	Lifecore	HA-200K
5-Methylfurfurylamine	TCI Chemicals	MFCD00143471
Methylcellulose	Spectrum Chemicals	ME137
Vitronectin peptide	Synthesized in house	N/A
MMP-degradable cross-linker	Synthesized in house	N/A
Silica beads	VWE	CA75873-698
Hyaluronidase	Sigma-Aldrich	H3884
Cell culture reagents		
Matrigel	Corning	354230
Dispase	Stem Cell Technologies	7913
Medium 231	Thermo Fisher Scientific	M-231-500
Smooth Muscle Growth Supplement	Thermo Fisher Scientific	S00725
Trypsin	Thermo Fisher Scientific	25300062
Collagenase from Clostridium histolyticum	Sigma-Aldrich	C0130
Ethyl 3-aminobenzoate methanesulfonate (Tricaine)	Sigma-Aldrich	E10521
Accutase	Stem Cell Technologies	7920
Essential 8 components		
DMEM/F12	Thermo Fisher Scientific	11330107
L-ascorbic acid-2-phosphate magnesium	Sigma-Aldrich	A8960
Sodium selenite	Sigma-Aldrich	S5261
FGF2	Thermo Fisher Scientific	PHG0263
Insulin	Wisent Bio Products	511-016-CM
Sodium bicarbonate	Sigma-Aldrich	S5761
Transferrin	Sigma-Aldrich	T0665
TGFβ1	Thermo Fisher Scientific	PHG9202
Commercial kits and materials		
VEGF-D DuoSet ELISA Kit	R&D Systems	DY622
BOLT 4-12% 1 mm, 10-well gel	Thermo Fisher Scientific	NW04120BOX
Stain-Free 4–20% 1mm 15-well gel	Bio-Rad	4568096
NucleoSpin® RNA 740955.250 D-Mark Bio	Machery-Nagel	740955.250
Cell models		
H9 human embryonic stem cell, WT	WiCell	WB67614
H9 *TSC2*$^{-/-}$	Generated in house [1]	N/A

H9 teratoma-derived LAM cells (WT and *TSC2*$^{-/-}$)	Generated in house	N/A
Animal models		
NOD.Cg-*Prkdc*^{scid} *Il2rg*^{tm1Wjl}/SzJ (NSG)	Jackson Laboratory	005557
casper mutant zebrafish	Gift from Dr. Leonard Zon [2]	N/A
Software and algorithms		
ImageJ 1.53c	ImageJ	N/A
R 4.0.3	R	N/A
RStudio 1.3.1093	RStudio	N/A
Prism 9	GraphPad	N/A
Gene Set Enrichment Analysis (GSEA)	[3]	N/A
Mechanism of Action Miner (Elion™)	BioSymetrics	Described here

Cell culture

Pluripotent stem cell culture

H9 hPSCs (female) were maintained on a thin layer of 0.16 mg/mL Matrigel at 37°C, 10% CO_2. Cells were fed with Essential 8 media, prepared in house. Cells were passaged by incubation with 500 μM EDTA for 3 min., then cell scraping and transfer to a new pre-coated plate by wide-bore pipette.

E8 media recipe

E8 media was made in house by mixing the following components: DMEM/F12 (as the base solvent media), L-ascorbic acid-2-phosphate magnesium (64 mg/L), sodium selenium (14 μg/L), FGF2 (100 μg/L), insulin (19.4 mg/L), $NaHCO_3$ (543 mg/L) and transferrin (10.7 mg/L), and TGFβ1(2 μg/L). Osmolarity of all media was adjusted to 340 mOsm at pH 7.4 using either NaOH (10 M) or HCl (10 M). All the media were stored at 4°C and were used within 2 weeks of production. L-ascorbic acid-2-phosphate magnesium is the stable form of L-ascorbic acid in cell culture.

LAM and control cell model derivation and culture

LAM cell models were established via a previously reported *in vivo* differentiation protocol of human pluripotent stem cells.[4] We differentiated a previously reported pair of mCherry$^+$ WT and genome-engineered *TSC2*$^{-/-}$ hPSCs, derived from the H9 parental lineage (female cells).[1] First, we generated teratomas in female NOD.Cg-*Prkdc*^{scid} *Il2rg*^{tm1Wjl}/SzJ (NSG) mice as described in **Mouse teratoma formation** section. At end point, mice were euthanized and dissected under sterile conditions. The teratomas were extracted while carefully ensuring minimal mouse tissue remnants. The teratoma was minced and then rotated in a 5 U/mL Dispase solution at 37°C for 30 mins. Digested tissue was plated on a thin layer of 0.16 mg/mL Matrigel at 37°C, 5% CO_2 in Medium 231 containing Smooth Muscle Growth Supplement. Tissue clumps were removed the following day. The remaining monolayer was expanded and passaged by treatment with 0.05% Trypsin for 5 min. Maintenance culture conditions included a thin layer of 0.16 mg/mL Matrigel at 37°C, 5% CO_2 in Medium 231 containing Smooth Muscle Growth Supplement. Cells were expanded for two passages before cryopreservation and use in subsequent experiments at passages 3-5.

LAM and control cell model clonal isolation

LAM cells were clonally isolated by limiting dilution. Briefly, bulk cell cultures were dissociated and serially diluted to a concentration of ~ 0.3 cells / 100 µL. We used this concentration to optimize number of single cells isolated while minimizing two or more cells contributing to a single clone. We added 100 µL of the suspension to each well of a 96-well plate containing a thin layer of 0.16 mg/mL Matrigel. Clones were expanded for 10 days before dissociating and plating onto the hydrogel.

Hydrogel culture

Reagent production

Hydrogel culture was conducted according to a previously established protocol.[5] Briefly, a hyaluronic acid polymer backbone was derivatized with 5-methylfurfurylamine to 65% substitution (confirmed by ^{1}H NMR). A vitronectin-mimetic peptide (maleimide)-KGGPQVTRGDVFTMPG, and MMP-degradable peptide crosslinker (maleimide)-KKGRGPQGIWGQKGPQGIWGQ-K(maleimide)S were synthesized using microwave-assisted Fmoc solid phase peptide synthesis with a CEM Liberty Blue automated peptide synthesizer. Hydrogel viscoelasticity was increased by incorporation of methylcellulose derivatized with reactive thiol groups.

Hydrogel gelation and culture

All chemically synthesized hydrogel components were mixed to the following final concentrations: 0.9 % methylfuronated hyaluronate, 2.3 mM MMP crosslinker, 100 µM vitronectin peptide, and 0.05 mg/mL thiolated methylcellulose. 15 µL of the solution was added to each well of a 384-well plate and permitted to gel at 37°C for 3 hours. Following gelation, wells were hydrated with PBS and then subjected to three media washes interspaced with incubations at 37°C for 45 mins. LAM or control cells were then dissociated, added to plates containing hydrogel, and spun for 3 min. at 10*g* to achieve immediate contact with the hydrogel. We added 2,000 cells per well in a 384-well plate format, and 10,000 cells per well in a 96-well plate format.

Cell treatments

Drug treatments

All small molecule compounds were diluted in either DMSO or PBS, unless otherwise stated. The appropriate diluent-matched vehicle control was included in every experiment. Drugs were added as 5X stock concentrations to wells already containing cells, at a volume of 25% of the media volume already in the well, thereby achieving a desired 1X final concentration. This was performed to avoid full media exchanges upon drug introduction, which detaches cells in our sensitive miniaturized plate formats. Rapamycin was consistently used at a 20nM concentration. All compound treatments were conducted for 72 hours unless otherwise stated.

Live cell staining

Live cell staining dyes were used at the following final concentrations: 10 μg/mL Hoechst 33342, 50 nM SyTOX Green, and 4 μM Caspase-3 Enzyme Substrate. Cells were incubated in the dyes for 30 min. prior to imaging. To avoid cell detachment in the miniaturized well format, live imaging dyes were not washed prior to imaging; this did not impact image acquisition as dyes are minimally fluorescent unless bound to the target molecule.

Cytotoxicity-invasion assay

Cells were permitted to invade through the hydrogel (384-well format) for 72 hrs while incubated at 37°C and 5% CO_2. At end point, Hoechst and SyTOX were added directly to all wells as described in **Cell treatments** section. Whole-well multi-planar images were acquired by widefield microscopy with 40 μm separation between z-stacks. Following image acquisition, wells were fixed overnight in 10% formalin. We then added 1μg of silica beads to each well and acquired multiplanar brightfield images, with the plane of maximal contrast used to determine

hydrogel-liquid interface (described in **Image analysis**). Acquiring location of the hydrogel interface (i.e., start of the cellular position) is essential for accurate invasion distance calculation; the hydrogel exhibits a meniscus which leads to a variable Z starting position depending on the XY location.

Microscopy

We employed a high content imager (Thermo Fisher Scientific, Arrayscan VTI) to acquire multi-well and multi-planar images. Whole-well images (384-well plate format) of cells invading through hydrogel, stained with live cell dyes, were acquired by widefield microscopy with 40μm interval z-stacks. Unstained cells were imaged using a brightfield module. Tiled images of cells grown and stained on plastic were also acquired by high content widefield microscopy. Rodent subcutaneous xenografts were visualized by *in vivo* imaging (PerkinElmer, IVIS®). Zebrafish larvae xenografts were imaged by epifluorescence widefield microscopy (Zeiss, AxioObserver 7). Image analysis methods are reported in the **Image analysis** section.

Three-dimensional drug screen

Screen design

Both WT and *TSC2*$^{-/-}$ cells were treated with every drug from the Ontario Institute for Cancer Research (OICR) Kinase Inhibitor and Tool Compound library (total of 800 compounds) at a concentration of 5 μM ± 20nM rapamycin. Only one technical replicate of each drug was tested in the initial screen. Cells were treated for 72 hr. while cultured in hydrogel and assessed at end point for cytotoxicity and invasion modulation as described in **Cytotoxicity-invasion assay**. Each plate included internal vehicle-treated only controls. Z' was calculated for

cytotoxicity and invasion modulation using vehicle-treated samples (negative control), 10 µM Y27632-treated (positive control, invasion), and 5 µM Carflizomib-treated (positive control, cytotoxicity). To achieve the throughput necessary for a therapeutic screen, we acquired live cell images by high content microscopy paired with automated image analysis tools developed in house.

Compound score calculation

To identify drugs with statistically significant effect(s), we computed z-scores for invasion modulation, cytotoxicity, selective invasion modulation, and selective cytotoxicity, for each WT and *TSC2*$^{-/-}$ in the presence or absence of rapamycin. We confirmed that the reference population of vehicle-treated controls for each metric was normally distributed and variance did not vary with effect mean. Cytotoxicity is determined by the percentage of SyTOX^{+} cells; invasion modulation is determined by the percentage of cells invading past the vehicle-control median threshold (See **Image analysis** section). Selective cytotoxicity is determined by the difference in cytotoxicity between WT and *TSC2*$^{-/-}$ cells, where positive values indicate more dead cells in the *TSC2*$^{-/-}$ condition. Selective invasion modulation is determined by the difference in cytotoxicity between *TSC2*$^{-/-}$ and WT cells, where positive values indicate fewer invading cells in the *TSC2*$^{-/-}$ condition. We then calculated p-values and corrected for multiple hypothesis testing by computing false discovery rates. All computation was performed using R 4.0.3 and RStudio 1.3.1093.

Target enrichment analysis

To refine our candidate compound list, we performed target enrichment analysis using a modified version of the GSEA algorithm.[3] Enrichment analysis was performed separately for cytotoxicity and invasion modulation. For cytotoxicity, we focused our compound list to drugs that showed selective cytotoxicity, either in the presence or absence of rapamycin. If a drug was shown to be significantly beneficial in one condition but significantly detrimental in the other, it was excluded. We then derived a singular compound score by computing the arithmetic mean across the two conditions. Similarly, for invasion modulation, we focused our compound list to drugs that exhibit anti-invasion effects towards WT or *TSC2*$^{-/-}$ cells, either in the presence or absence of rapamycin. Again, we excluded compounds that showed opposing effects, and derived a singular compound score by arithmetic mean across conditions.

We next generated a background target list using known compound targets as annotated by the OICR. We created generalizable categories wherever possible, however, there were many targets that could not be grouped and conferred an n = 1 category. To avoid the possibility of bias, these categories were established by an independent author blinded to the original compound results. Using this background list and our compound score lists as described above, we determined target enrichment using the GSEA algorithm.[3]

Elion™ analysis

A limitation to our analyses is the small number of compounds which were identified to selectively eliminate *TSC2*$^{-/-}$ cells. We sought to extend our compound list *in silico* using a structure-based approach with Elion™ (Mechanism of Action Miner), conducted by an independent group. In brief, chemical features are extracted from compound structures and matched with screen performance values to train a machine learning algorithm for prediction of

other possibly efficacious compounds. Compounds predicted to be efficacious *in silico* are then analyzed by target enrichment and pathway analysis.

In more technical detail, Elion™ is a software package that ingests binary phenotypic data linked to individual drug treatments to suggest possible underlying protein targets and molecular pathways. The platform inputs phenotypic screening data in the form of a two-column CSV file corresponding to the chemical structure in SMILES format alongside a binary bioactivity reading.

Using this dataset, a total of 8,000 features are generated for each supplied chemical structure. These features are comprised of chemical fingerprints (ECFP4, FCFP4, RDK-layered fingerprint, and MACCS) alongside physical properties (e.g., molecular weight, total polar surface area, LogP). If there are fewer than 8,000 rows in the input data set, a subset of features are chosen for downstream machine learning. The size of this feature subset is set to be 70% of the number of rows in the input data set. Feature selection is performed using a bootstrapped logistic regression strategy. In brief, a set number of features are sampled from the original feature set and are used to train a logistic regression model. The coefficients of this model are then used to rank feature importance. This process is repeated 5,000 times and the resulting coefficients are averaged for each feature to create a summarized feature importance score.

Once a feature set is chosen, a total of 6 machine learning models are built and evaluated on the input data set (XGBoost, random forest, Gaussian naive Bayes, uniform and distance weighted K-nearest neighbours, and Gaussian process classifiers). Each model is trained and evaluated using 10-fold cross validation while recording classification performance according to accuracy, ROC-AUC, precision and recall. The best performing model is then chosen and used

to rank a set of 1 million compounds (curated from public databases) according to probability of inducing the given phenotype.

Of the 1M ranked compounds, several are annotated according to experimentally validated protein targets and mechanisms of action (MoA). The GSEA algorithm is used to determine which of these targets and MoAs are most positively enriched within the ranked set of compounds. We then subset this list using an FDR threshold to identify a set of enriched targets and MoAs. Using the enriched targets, we perform gene ontology and protein family pathway enrichment using the Fisher exact test. A Bonferroni corrected p-value threshold of 0.05 is used to identify cellular pathways corresponding to the phenotype of interest.

As a result of this process, Elion™ translates an input phenotypic screen into three informative outputs. First, it supplies a ranked list of publicly available compounds prioritized according to their likelihood to induce the given phenotype. Second, it provides a list of targets and MoAs likely to mitigate the provided phenotype. Last, it annotates these targets with enriched cellular pathways. All of these results are presented in a web application annotated with rich descriptions and link-outs to relevant genetic databases.

Image analysis

Identification of cell spatial positions and cell invasion distance

We identified XYZ cell positions in the hydrogel by analysis of the Hoechst channel z-stack. We first determined XY positions by employing the ImageJ 1.53c "Find maxima" function on the z-stack maximum intensity projection. We automated the determination of the noise (or background) threshold by empirical iteration. Using the assumption that true Hoechst signal should be substantially above background fluorescence, we computed "Find maxima" with a

liberal threshold, and then progressively increased threshold stringency until the number of identified points did not vary with each stepwise threshold change. Each maxima was determined to correspond to a single cell spatial location. Following, for each XY spatial position, we iterated through the Hoechst z-stack and identified to the point of maximal intensity, corresponding to the cell Z position.

To determine the invasion distance of each single cell, we must first know the cell starting position, which varied across XY positions due to the meniscus exhibited by the hydrogel. To identify determine hydrogel interface Z position across the XY plane, we used the silica bead brightfield images (described in Cytotoxicity-invasion assay). For each XY cell spatial position, we iterated through the brightfield z-stack and identified the point of maximal contrast, which corresponded to the layer containing silica beads (due to diffraction). We then determined individual cell distances travelled by computing the difference between cell starting and final positions. This process, automated for high throughput analysis, was scripted in ImageJ 1.53c.

Binarization of live cell stains

Live cell stains (i.e. SyTOX and Caspase-3 enzyme substrate) were binarized into a positive or negative signal for each cell. We first created a masking around the Hoechst signal of each cell in the maximum intensity projection image, then measured the total fluorescent signal of the live cell stain within each masking. To binarize in an automated fashion, we fit an empirical probability density function (ePDF) by kernel density estimation on the vehicle control sample values. Assuming the majority of untreated samples should be negative for cell death stains, we determined the threshold for binarization to by the first local minimum of the negative

control ePDF. Cells across conditions were then binarized according to their matched control threshold. This process, automated for high throughput analysis, was scripted in ImageJ 1.53c and R 4.0.3 within the RStudio 1.3.1093 environment.

Quantification of cellular invasion

Cellular invasion was determined by number of cells invading past a fixed distance. As the invasion distance varied slightly batch to batch (see Fig. 1F), distance thresholds for each experiment were based on within-experiment vehicle controls. We used both the median invasion of vehicle controls, which is sensitive to detecting decreases in invasion, and 90th percentile invasion of vehicle controls, which is sensitive to detecting increases in invasion. Genotype-specific thresholds were employed due to the differing invasion distances between WT and *TSC2*$^{-/-}$ cells. To determine invasion of alive cells only, SyTOX^{+} positive cells were removed from the distribution prior to calculation of invasion percentages. This process, automated for high throughput analysis, was scripted in R 4.0.3 within the RStudio 1.3.1093 environment.

Immunofluorescence stain quantification

Immunofluorescence experiments were quantified using the raw image files, ensuring the absence of detector saturation. We created a masking around the Hoechst signal of each cell in the maximum intensity projection image, then measured the mean fluorescent signal of the protein-of-interest within the total masking area. Measurements per replicate were re-scaled from 0 – 1 by dividing by the replicate maximum value. We note that comparisons between plastic and hydrogel samples cannot be directly made, as the imaging parameters differ between the two-dimensional vs. three-dimensional environment (see Fig. 2B).

Invasion quantification upon zebrafish xenotransplantation

Invasion of transplanted $mCherry^+$ cells was performed in a semi-automated fashion on blinded images (Fig. S6B). For each image, a region of interest was manually selected on the maximum intensity Z projection, to distinguish areas with $mCherry^+$ cells from surrounding auto-fluorescent regions (e.g., zebrafish eye, yolk sac, ossicle). Images were then binarized using a constant threshold to distinguish positive signal from background. Pixels were classified into "invaded" or "not invaded" based on the distance from the center of the initial injection site. We used the average of the first local minima of the positive pixel histogram from 1 day post injected images to determine the distance for classification of invaded or not. Following pixel classification, the ratio of the total positive pixel intensity in each group was computed to determine the zebrafish invasion score. Groups were then unblinded and graphed. This process was scripted in ImageJ 1.53c and R 4.0.3 within the RStudio 1.3.1093 environment.

RNA-seq

RNA extraction and quality control

Extraction of RNA from cells embedded in hydrogel is made challenging by the low cellular density relative to the abundant extracellular matrix. To extract RNA, we developed an extraction protocol that combines phenol-chloroform phase separation with column-based purification. We first added TriZOL directly to wells and homogenized the cell-hydrogel mixture using a 26-gauge needle. We then centrifuged the lysate for 5min, 12,000*g*, at 4°C to pellet the cross-linked hyaluronic acid matrix. We extracted the supernatant and mixed in chloroform, followed by centrifugation to induce phase separation. The colourless aqueous phase was

extracted and mixed with equal volumes of 70% EtOH. Following a brief incubation at room temperature, the solution was eluted through a Machery-Nagel Nucleospin column. We proceeded with column-based purification as per manufacturing protocol.

RNA-sequencing and raw data processing

RNA samples were shipped to the Donnelly Sequence Centre (Toronto, Canada) for RNA quality-control, library preparation, and next-generation sequencing. RNA integrity was assessed via Bioanalyzer (Agilent) and only samples with RIN > 8 were prepared for sequencing. Oligo(dT) priming via SMART-Seq v4 (Takara Bio) preparation kit was used to generate full-length cDNA libraries. Samples were subjected to paired-end sequencing on a NovaSeq 6000, 100c (Illumina) to a depth of ~50 million reads per sample.

Raw sequence read quality was assessed by *FastQC*. Read feature assignments and duplication rates were determined using *featureCounts* and *Picard*. Overall mapping rate was assessed with *HISAT2*. Finally, read assignment to transcripts was performed using *Salmon*, generating a final pseudocount abundance matrix. All QC processing was summarized using *MultiQC* and programmed in R 4.0.3 within the RStudio 1.3.1093 environment. RNA-seq data is accessible at the Gene Expression Omnibus (GEO) repository with accession GSE179044.

Differential gene expression and enrichment analysis

Pseudocount abundance data generated by *Salmon* was imported into the *DEseq2* framework in R for differential expression and enrichment analysis. Principal components analysis was conducted on all samples to visualize transcriptomes in a two-dimensional space. For single variable differential expression testing, we subsetted samples to only include the

untreated and fit the following model: ~ *batch* + *genotype* + *substrate*. We then tested for genes with significant coefficients by Wald test, separately for genotype and for culture substrate. To assess for changes across genotype that differs between matrix condition, we again subsetted for untreated samples and fit the following model: ~ *batch* + *genotype* + *substrate* + *genotype:substrate*. The interaction term coefficient for each gene was tested for significance by Wald test. Differentially expressed genes were called when false discovery rate (FDR) < 0.05, ± |log2FoldChange| > 1 (as indicated in the text).

To visualize expression values by heatmap or gene cluster, sample conditions were collapsed by abundance summation, normalized, and then transformed by regularized $\log_2$ transformation (implemented in *DEseq2*). Heatmaps were generated using the *pheatmap* package in R and gene clusters were generated by hierarchal clustering. GO term enrichment was performed using *clusterProfiler* on significant DEGs (FDR < 0.05, ± |log2FoldChange| > 1). All analysis was conducted using R 4.0.3 within the RStudio 1.3.1093 environment.

Animal studies

Mouse teratoma formation

hPSCs were dissociated into single cells by Accutase treatment for 15 min at 37°C. Single cells were harvested, washed, and resuspended in 5 mg/mL Matrigel. Female 8-week-old NSG mice were treated with buprenophrine 1 hour before injection, then anesthetized by isofluorane under a continuous stream of O_2. We bilaterally injected $1x10^6$ hPSC into the mouse tibialis anterior. We allowed teratomas to grow over a 12-week period, after which mice were sacrificed and teratomas extracted.

Mouse subcutaneous xenografts

LAM cells were dissociated into single cells by 0.05% Trypsin treatment for 5 min. at 37°C, washed, and resuspended in 5 mg/mL Matrigel. Female 8-week-old NSG mice were anesthetized by isofluorane under a continuous stream of O_2 and injected with $1x10^6$ cells subcutaneously in each rear flank. We monitored for palpable tumor growth weekly over a four-month period, after which animals were sacrificed.

Mouse IVIS image acquisition

Female 8-week-old NSG mice that were injected with LAM cells in each rear flank were monitored for tumor growth by endogenous mCherry expression of LAM cells. Mice were anesthetized by isoflurane under a continuous stream of O_2 and shaved to eliminate background fluorescence from the fur coat. Mice were then imaged at fixed exposure times by *in vivo* imaging (PerkinElmer, IVIS®).

Zebrafish toxicity assay

72-hour post-fertilization (hpf) zebrafish larvae were arrayed one larva per well in a 96-well plate and treated with increasing concentrations of each inhibitor for 72 hrs to ascertain toxicity thresholds. There were no *in vivo* toxic effects at the experimental *in vitro* concentrations and thus, zebrafish experimental doses were chosen to stay consistent with *in vitro* treatment doses.

Zebrafish hindbrain ventricle xenotransplantation

For each injection experiment, a separate cryovial of cells was thawed and cultured 3 days prior to zebrafish transplantation, without any subculturing. On the day of transplantation, cells were dissociated by 0.25% trypsin, centrifuged for 5 mins at 300*g*, and resuspended in approximately 30 µL of culture medium for injection. 72 hpf zebrafish larvae were anesthetized with 0.09 mg/mL tricaine (Millipore Sigma) and arrayed in troughs of an agarose injection plate and used for cell transplantation using protocols described previously.[6,7] The cells were backloaded into a pulled capillary needle and allowed to settle for approximately 20 mins at 35°C to ensure a cell pellet at the bottom of the needle. A PLI-100A Pico-liter Microinjector (Warner Instruments) was used to manually inject 50-100 cells into the hindbrain ventricle (HBV) of each larva. Following injections, the larvae were kept at 35°C for the remainder of the experiment.

Zebrafish drug treatments

1 day post injection (dpi), injected larvae were screened on an Axio Observer 7 fluorescent microscope under an mCherry filter to ensure the presence of cells only in the HBV. Groups of 20-30 positively injected larvae were randomized into groups to be treated with either vehicle control (DMSO), 20 nM rapamycin alone, 5 µM SB939 alone, 20 µM SAHA alone, 1 µM LBH589 alone or with one HDACi in combination with rapamycin by immersion therapy for 72hrs. At the experimental endpoint (3 days post-treatment) the groups of larvae were blinded and imaged on the Axio Observer 7 using the z-stack function to capture cell movement in all planes. Blinded groups of images were then subjected to automated invasion analysis by an independent study author.

Zebrafish whole larval dissociation and fixation

At 1 dpi (baseline) and 4 dpi (three days post-treatment), 20 larvae from each group were euthanized and dissociated in 100mg/mL collagenase solution for approximately 30 mins. Upon completion of dissociation (i.e., single cell suspension formed), 200 µL of 100% FBS was added to slow the enzymatic reaction. The samples were then centrifuged for 5 min. at 300 *g* and the supernatant was removed, leaving a pellet of human tumor cells among the zebrafish cells. The samples were washed once in 30% FBS in PBS and centrifuged once more for 5 min. at 300 *g*. The supernatant was removed and 250µL of 4% PFA in PBS was added to each sample for 20 min. in the dark. 1mL of PBS was added and samples were centrifuged and PFA supernatant was removed. Samples were resuspended in 500 µL PBS, stored at 4°C, and blinded prior to flow cytometry analysis.

Immunofluorescence staining

The following protocol is for immunofluorescence staining of cells in monolayer culture on plastic. Modifications for whole-mount (WM) staining of cells in three-dimensional hydrogel are indicated throughout.

Cells were fixed with 4% PFA for 15 min. (WM: 30 min.) at room temperature. Wells were washed 3 x 5 min. (WM: 20 min.) with PBS, then permeabilized with 0.1% Triton-X in PBS for 20 min. (WM: 40 min) at room temperature. Wells were washed 3 x 5 min. (WM: 20 min.) with PBS, then blocked with 1% BSA in PBS for 1 hr. (WM: 2 hr.) at room temperature. We then added primary antibody diluted in blocking solution for overnight incubation at 4°C. The following concentrations of antibodies were employed: PMEL (1:50), ACAT2 (1:100), pS6RP$^{Ser235/236}$(1:100), and p4E-BP1$^{Thr37/46}$(1:200). The next day, wells were wash 3 x 5 min.

(WM: 5 x 30 min.) with PBS, then incubated with fluorescent secondary antibodies diluted blocking solution for 1 hr. (WM: 2 hr.) at room temperature. All fluorescent secondary antibodies were used at a 1:1000 dilution. Wells were then washed 3 x 5 min. (WM: 5 x 30 min.) with PBS then counterstained with 10 µg/mL Hoechst 33342 for 30 min. (WM: 45 min.) Wells were washed 3 x 5 min (WM: 5 x 30 min.), then mounted with a 90% glycerol (WM: PBS, as the hydrogel disintegrates in glycerol) solution made in house, prior to imaging.

Enzyme-linked immunosorbent assay (ELISA)

Maintenance cultures of cells at equivalent densities were incubated for 16 hr. in Medium 231 ± 20nM rapamycin, without serum supplement. Conditioned media was collected and centrifuged to remove any cellular debris, then assayed by VEGF-D ELISA kit (R&D Systems, DY622) following the manufacturer protocol.

Flow cytometry

LAM cells were dissociated into single cells by 0.05% Trypsin treatment for 5 min. at 37°C, washed, and then fixed with 4% PFA for 15 min. at room temperature. Fixing solution was diluted out 1/10 in PBS, cells were pelleted by centrifugation, and supernatant discarded. For details on zebrafish single cell preparation, see Zebrafish whole organism dissociation and fixation section. Fixed single cell suspensions were permeabilized with 0.1% Triton-X in PBS for 20 min. at room temperature. Permeabilizing solution was diluted out 1/10 in PBS, cells were pelleted by centrifugation, and supernatant discarded. Samples were then blocked with 1% BSA or 5% Goat Serum in PBS for 1 hr. Cells were pelleted by centrifugation, supernatant discarded, and primary antibodies diluted in blocking solution were added for overnight incubation at 4°C.

The following concentrations of antibodies were employed: PMEL (1:50), ACAT2 (1:100), mCherry (1:1000), and Cleaved CASP3 (1:500). The next day, primary antibody solution was diluted out 1/10 in PBS, cells were pelleted by centrifugation, and supernatant discarded. Samples were then incubated with fluorescent secondary antibodies diluted blocking solution for 1 hr. at room temperature. All fluorescent secondary antibodies were used as a 1:1000 dilution. Secondary antibody solution was diluted out 1/10 in PBS, cells were pelleted by centrifugation, and supernatant discarded. Samples were next counterstained with 10 µg/mL Hoechst 33342 for 20 min. at room temperature. Hoechst 33342 solution was diluted out 1/10 in PBS, cells were pelleted by centrifugation, and supernatant discarded. Finally, cells were strained and analyzed using the LSRFortessa (BD) flow cytometer.

Low input western blot

Hydrogel culture must be performed in a miniaturized format to maintain the appropriate mechanics as previously reported.[5] Naturally, this poses a challenge for collecting sufficient protein for standard molecular biology methods, such as a western blot. To address this challenge, we developed a method for a low input western blot that includes in-well lysis and sample preparation, followed by a gel-based method for sample normalization.

Samples were cultured on plastic or in hydrogel for 72 hr. Following, sample media was aspirated to the hydrogel interface (leaving a similar volume in plastic wells) and an equivalent volume of 2X Laemmli-RiPA buffer was added to each well. Samples were incubated for 10 min. at 37°C and triturated up and down, careful not to disturb the hydrogel. The sample volume was then extracted and boiled for 10 min. at 70°C. As this extraction contains a large amount of non-cellular derived protein components (due to degradation of the hydrogel MMP-cleavable

crosslinkers), standard protein quantification by colorimetric methods (e.g., BCA, and Bradford) are not reliable. Instead, we performed total protein quantification on gel-separated samples. First, an aliquot of each sample was electrophoresed on a stain-free 4–20% 1 mm 15-well gel, along with a serial dilution of a sample of known concentration. The gel was then activated by UV exposure and total protein visualized by ChemiDoc Gel Imager (Bio-Rad). We then calculated individual sample concentrations by comparing against the within-gel standard curve, without including bands corresponded to the hydrogel MMP peptides.

After sample extraction and quantification, we analyzed samples following standard western blotting procedures. To maximize sample input, Thermo Fisher Scientific BOLT gels were used, which contain space for up to 60 µL of sample per lane. We first separated samples by SDS-PAGE using a BOLT 4-12% 1 mm 10-well gel and MES running buffer. Samples were transferred onto a 0.45 µm PVDF membrane overnight at 4°C by wet transfer with Towbin buffer (containing 0.025% SDS and 10% MeOH). The membrane was then blocked by 5% BSA in PBS-T (0.1% Tween-20) for 1 hr. at room temperature. Following, the membrane was incubated overnight at 4°C in primary antibodies diluted in blocking buffer at the following concentrations: pS6RP$^{Ser235/236}$(1:5000), p4E-BP1$^{Thr37/46}$(1:1000), S6RP (1:500), 4E-BP1 (1:1000), TSC2 (1:5000), and ACTB (1:5000). The membrane was washed 3 x 5 min. with PBS-T, and then incubated for 1 hr. at room temperature in fluorescent secondary antibodies diluted 1:10,000 in blocking buffer. The membrane was washed 3 x 5 min. with PBS-T and then imaged using Odyssey Gel Imager (LI-COR Biosciences).

EdU proliferation assay

Cells were pulsed with 5 µM of EdU for 3 hr. Subsequently, cells were fixed with 4% PFA for 15 min. at room temperature. Wells were washed 3 x 5 min. with PBS, permeabilized with 0.1% Triton-X in PBS for 20 min. at room temperature, then washed again 3 x 5 min. We prepared the click reaction by mixing the following components in the described order, in PBS, to the indicated final concentrations: 4 mM Cu_2SO_4, 5 µM Sulfo-Cy5-N_3, and 100 mM L-ascorbic acid. The click reaction mix was added to wells containing cells and incubated at room temperature for 30 min. Wells were washed 3 x 5 min. with PBS, counterstained with 10 µg/mL Hoechst 33342 for 30 min, washed again 3 x 5 min., and then imaged.

Clonogenic assay

Cells were plated on the hydrogel and treated with HDAC inhibitors at the designated concentration for 72 hr. Following treatment, wells were washed 3 x 20 min. with media to remove the drug from solution. The hydrogel was then solubilized by addition of 150 U hyaluronidase per 15 µL hydrogel and incubated for 1 hr. at 37°C. Following, 0.05% Trypsin was added to the wells for 10 min. at 37°C to dissociate cells. Wells were triturated and then plated on two-dimensional tissue cultures plates, in serial dilution. Cells were permitted to proliferate for 10 days, forming colonies from single cells. Following, wells were fixed with 4% PFA for 15 min. at room temperature, then washed 3 x 5 min. with PBS. Colonies were stained with 0.1% crystal violet for 1 hr. at room temperature, washed 3 x 5 min. with ddH_2O, air dried, and imaged.

Supplementary tables

Table S1. Differential gene expression analysis of bulk RNA-seq data, untreated samples only. (A) DEG analysis of genotype (*TSC2*$^{-/-}$ vs. WT), controlling for substrate covariate. (B) DEG analysis of substrate (hydrogel vs. plastic), controlling for genotype covariate. (C) DEG analysis of interaction between genotype and ECM.

Table S2. GO term enrichment analysis of DEG lists. (A) GO term enrichment in *TSC2*$^{-/-}$ vs. WT DEG list (FDR < 0.05, $|\log_2 FC| > 1$). (B) GO term enrichment in hydrogel vs. plastic DEG list (FDR < 0.05, $|\log_2 FC| > 1$). (C) GO enrichment of genotype:substrate interaction DEG list (FDR < 0.05).

Table S3. Three-dimensional drug screen raw data. (A) Compound information from Ontario Institute of Cancer Research kinase inhibitor and tool compound libraries. (B-C) Cytotoxicity and invasion modulation effects of compounds, (B) statistic descriptions and (C) raw data.

Table S4. Enrichment results via adaptation of GSEA. Results for statistics of (A) selective cytotoxicity, positive enrichment (i.e., selectively cytotoxic towards *TSC2*$^{-/-}$), (B) selective cytotoxicity, negative enrichment (i.e., selectively cytotoxic towards WT), (C) invasion modulation, positive enrichment (i.e., attenuate invasion), (D) invasion modulation, negative enrichment (i.e., potentiate invasion).

Table S5. Elion™ structure-based compound analysis. (A-B) Significantly enriched targets and mechanisms of action by (A) selective cytotoxicity towards *TSC2*$^{-/-}$ cells and (B) invasion attenuation. (C-D) Significantly enriched GO and PFAM terms (based on significantly enriched targets) by (C) selective cytotoxicity towards *TSC2*$^{-/-}$ cells and (D) invasion attenuation.

Supplementary movies

Movie S1. Brightfield Z-stack of WT invading through the hydrogel, counterstained with Hoechst.

Movie S2. Brightfield Z-stack of *TSC2*$^{-/-}$ invading through the hydrogel, counterstained with Hoechst.

Supplementary figures

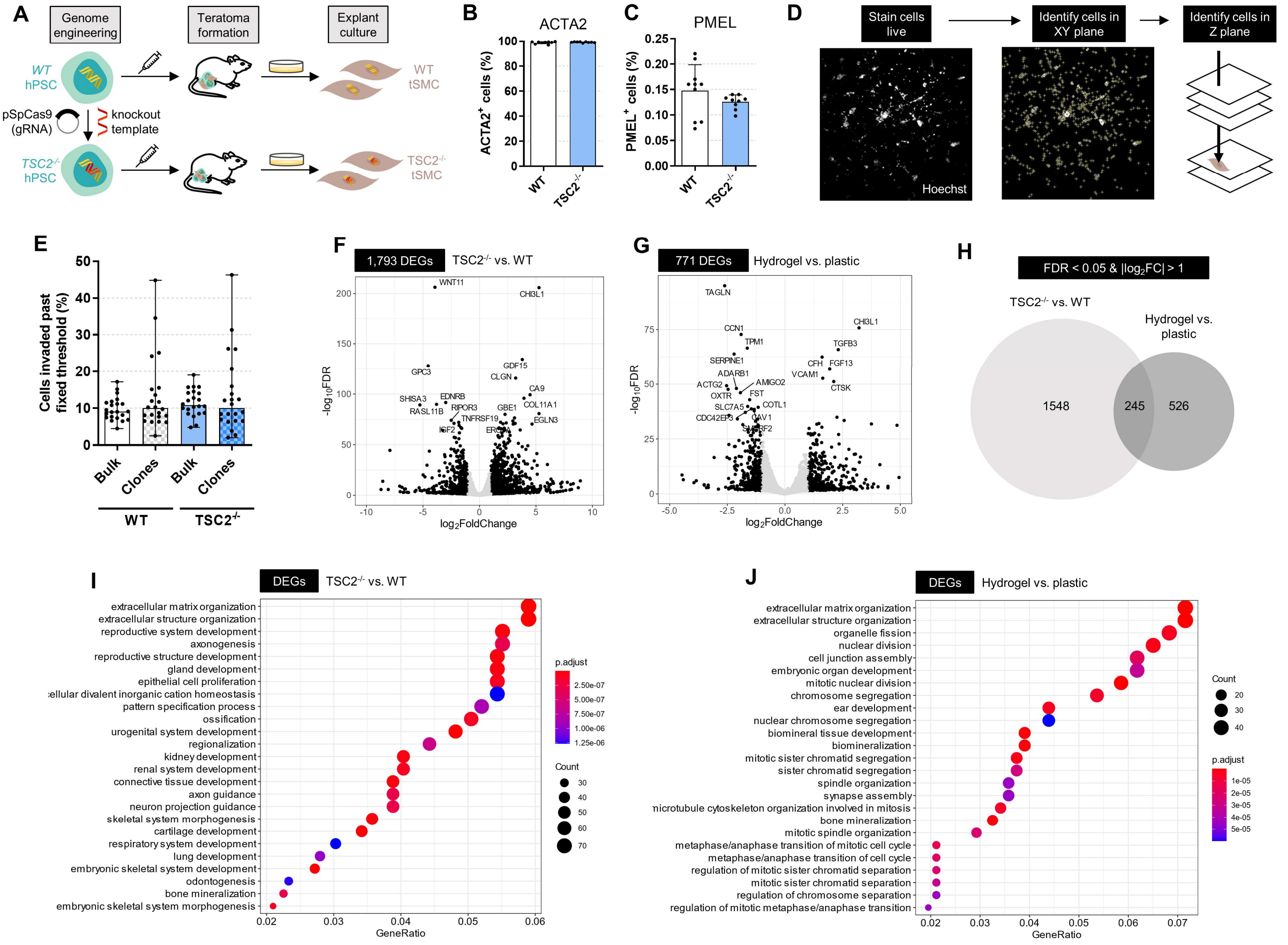

Figure S1. Hydrogel culture of stem cell-derived disease models exhibits features of LAM. (**A**) Schematic of generation of LAM cellular models. (**B-C**) Quantification of LAM markers by flow cytometry from cells in maintenance culture (mean ± SD; * = $p < 0.05$ by unpaired, two-tailed Student t-test; n = 10). (**D**) Schematic of cell position identification in XYZ planes. (**E**) Percentage of cells invaded past threshold set by 90th percentile invasion distance of bulk cultures, following three-day hydrogel culture. Bulk cultures are maintenance cultures of LAM cell lines; clones are populations of cells expanded from a single cell isolated from maintenance

cultures prior to seeding on hydrogel (mean ± data range; no statistical test). (**F-G**) Volcano plot upon comparing *TSC2*$^{-/-}$ vs. WT cells (F) and hydrogel vs. plastic samples (G). Points highlighted in black are considered differentially expressed (FDR < 0.05 and $|\log_2 FC| > 1$). The 20 most significantly DEGs are noted. (**H**) Overlap in DEG between genotype and culture substrate gene lists; genes considered as DEGs if FDR < 0.05 and $|\log_2 FC| > 1$. (I-J) Dotplot of GO term enrichment analysis of DEG lists (FDR < 0.05 and $|\log_2 FC| > 1$) upon comparing *TSC2*$^{-/-}$ vs. WT cells (**I**) and hydrogel vs. plastic samples (**J**). The 25 most significantly enriched terms are plotted.

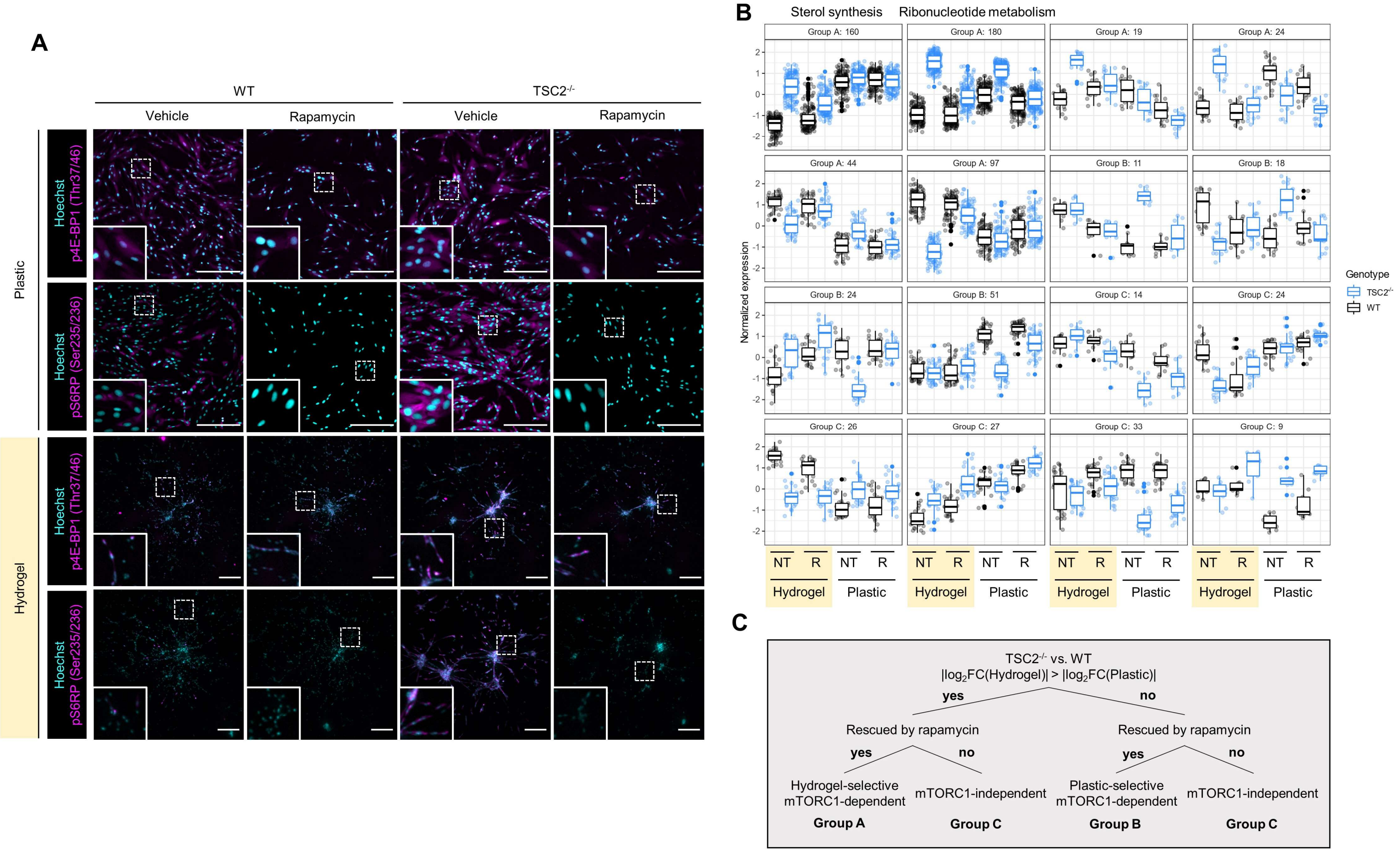

Figure S2. Hydrogel culture potentiates differential mTORC1-signalling between WT and TSC2$^{-/-}$ cells. (**A**) Representative maximum intensity projection images used for quantification, following culture on hydrogel or plastic for three days ± 20nM rapamycin. Scale bars of 250μm. (**B**) Gene clusters following hierarchal clustering of DEGs found significant (FDR < 0.05) in the interaction between genotype and ECM (761 genes). Clustering was based on the pattern of gene expression across the 8 employed conditions. The first two clusters are annotated to be enriched in sterol synthesis and ribonucleotide metabolism terms. (**C**) Classification scheme applied to the gene clusters visualized in (B). Labelling of Groups A, B, and C is for ease of visualization in (B) and does not confer any specific biological meaning.

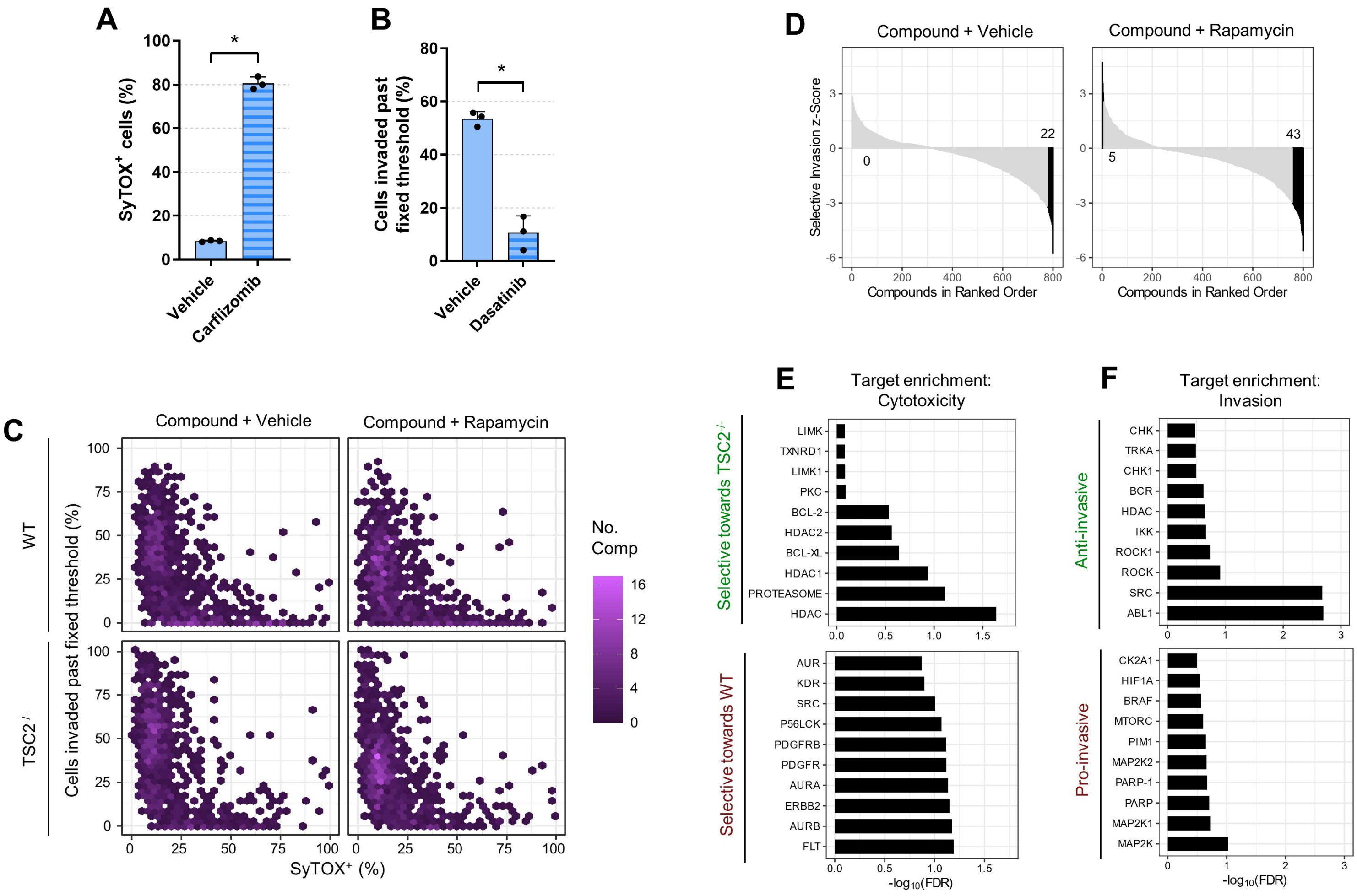

Figure S3. Three-dimensional drug screen identifies HDAC inhibitors as anti-invasive and selectively cytotoxic towards TSC2$^{-/-}$ LAM cells. (**A**) Percentage of SyTOX^{+} *TSC2$^{-/-}$* cells in hydrogel culture for three days ± 200nM carfilzomib (mean ± SD; * = $p < 0.05$ by unpaired, two-tailed Student t-test; n = 3). (**B**) Percentage of *TSC2$^{-/-}$* cells invaded past fixed threshold (determined by median invasion distance of untreated controls), following three-day hydrogel culture ± 40nM dasatinib (mean ± SD; * = $p < 0.05$ by unpaired, two-tailed Student t-test; n = 3). (**C**) Compound invasion modulation plotted against cytotoxicity, separated by genotype and rapamycin treatment. Fixed threshold determined by median invasion distance of genotype-specific untreated controls. Hexagonal plot employed to demonstrate compound densities. (**D**) Waterfall plots of compound selective invasion z-scores in ranked order; positive values indicate

greater anti-invasive effects towards *TSC2*$^{-/-}$, negative values indicate greater anti-invasive effects towards WT. Compounds conferring statistically significant selective invasion modulation highlighted in black. (**E-F**) Top 10 most statistically significant targets enriched in screen data, stratified by screen parameter. Enrichment analysis was performed via adaptation of the GSEA algorithm, using annotated targets of the compound library.

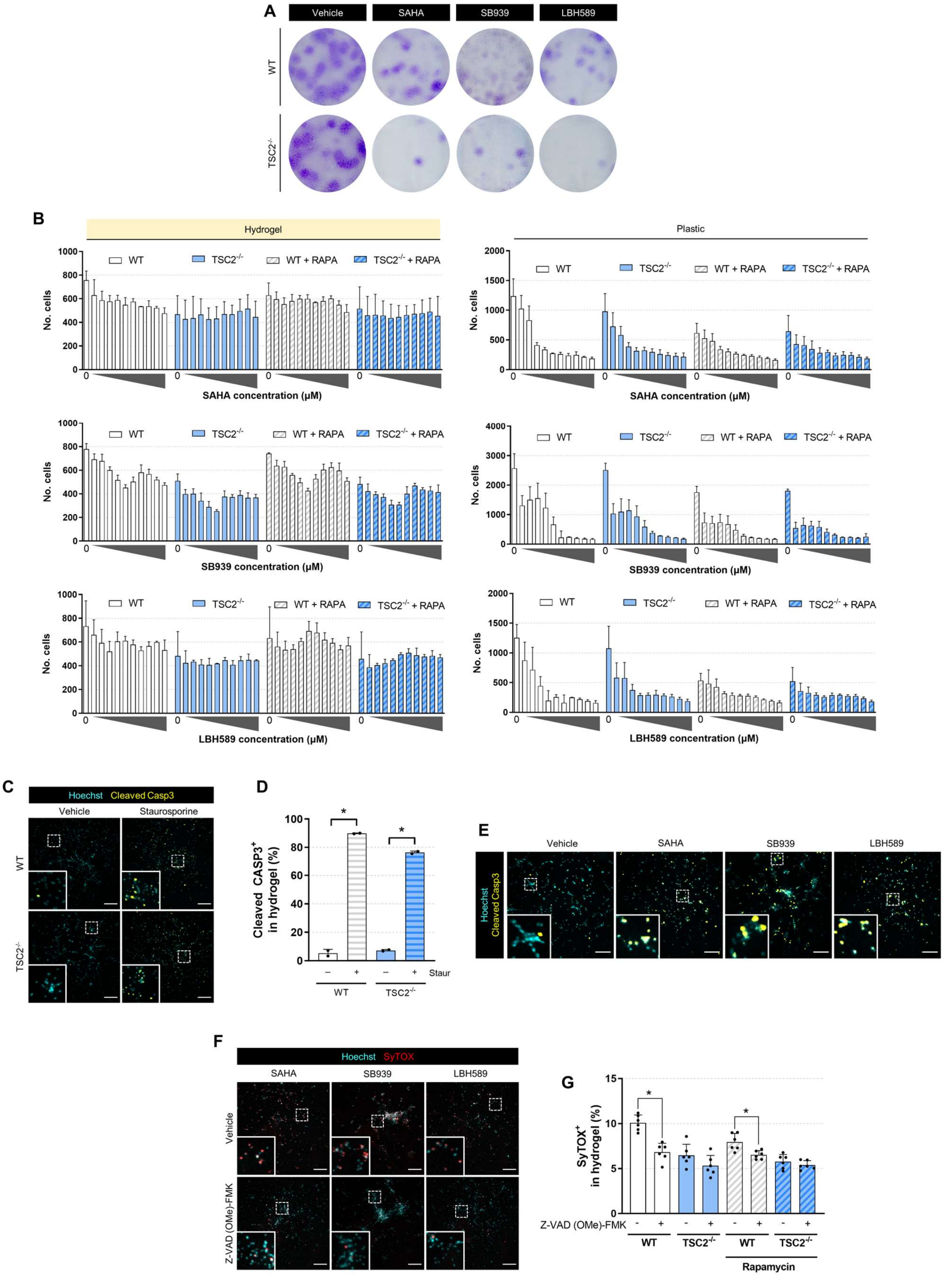
A
Vehicle
SAHA
SB939
LBH589
WT
TSC2-/-
B
Hydrogel
Plastic
WT
TSC2-/-
WT + RAPA
TSC2-/- + RAPA
No. cells
SAHA concentration (μM)
SB939 concentration (μM)
LBH589 concentration (μM)
C
Hoechst Cleaved Casp3
Vehicle
Staurosporine
WT
TSC2-/-
D
Cleaved CASP3+ in hydrogel (%)
Staur
WT
TSC2-/-
E
Vehicle
SAHA
SB939
LBH589
Hoechst
Cleaved Casp3
F
Hoechst SyTOX
SAHA
SB939
LBH589
Vehicle
Z-VAD (OMe)-FMK
G
SyTOX+ in hydrogel (%)
Z-VAD (OMe)-FMK
WT
TSC2-/-
WT
TSC2-/-
Rapamycin

Figure S4. Three safe-in-human HDAC inhibitors induce mTORC1-dependent selective cytotoxicity exclusively in hydrogel culture. (**A**) Clonogenic assay following three-day HDAC inhibitor treatment (20μM SAHA, 5μM SB939, 1μM LBH589) of cells cultured in hydrogel. After treatment, cells were extracted from hydrogel and replated in 2D to assess clonogenicity. (**B**) Number of cells detected in culture by high content imaging following three-day HDAC inhibitor treatment in hydrogel or plastic culture ± 20nM rapamycin. Inhibitor concentrations escalated in two-fold increments: SAHA (0.31μM min, 160μM max), SB939 (0.04μM min, 20μM max), and LBH589 (0.02μM min, 10μM max). Mean ± SD, n = 3. (**C-D**) Representative maximum intensity projection images and quantification of live cleaved caspase 3 dye used in hydrogel culture, following 4hr treatment of 1μM staurosporine (mean ± SD; * = $p < 0.05$ by one-way ANOVA with Bonferroni post-hoc comparisons; n = 2). Scale bars of 250μm. (**E-F**) Representative maximum intensity projection images of live cell imaging dyes used in hydrogel culture, following three-day treatment with HDAC inhibitors (20μM SAHA, 5μM SB939, 1μM LBH589) ± 25μM Z-VAD (OMe)-FMK. Scale bars of 250μm. (**G**) Percentage of SyTOX^{+} cells following three days in hydrogel culture ± 25μM Z-VAD (OMe)-FMK (mean ± SD; * = $p < 0.05$ by one-way ANOVA with Bonferroni post-hoc comparisons; n = 4-6).

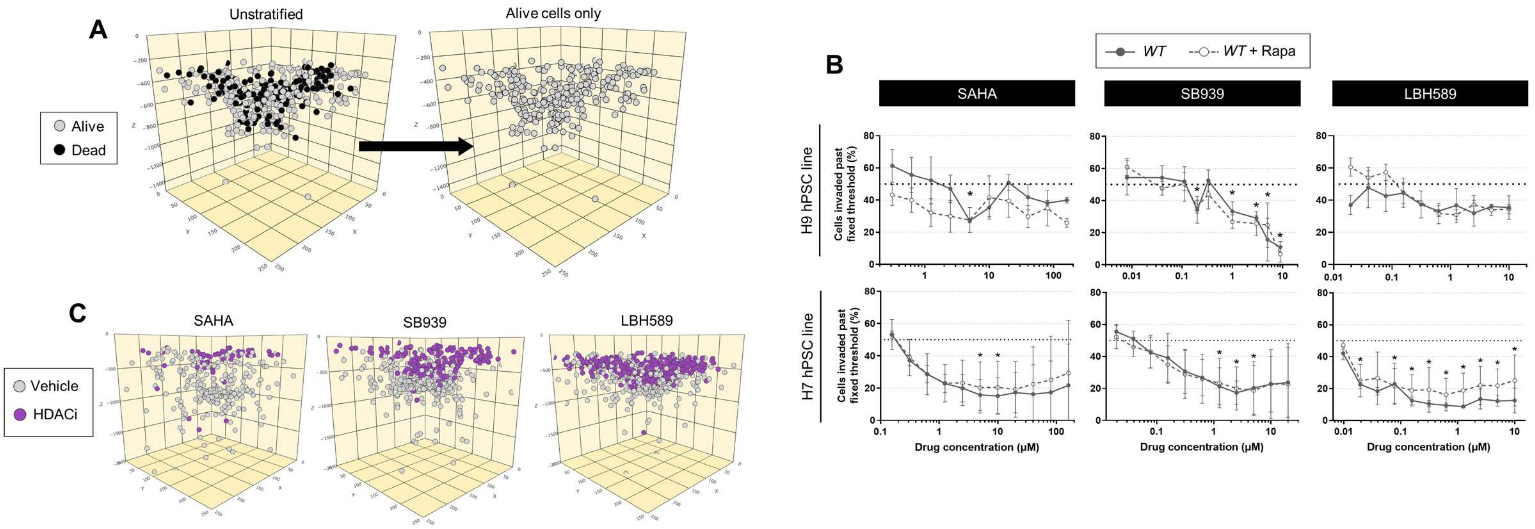

Figure S5. HDAC inhibitors attenuate cell invasion independent of cytotoxicity. (**A**) Schematic for removal of SyTOX^{+} cells to determine invasion distribution of live cells. (**B**) Live WT cells (H9 top panels, H7 bottom panels) invaded past fixed threshold set by median invasion distance of vehicle control, upon three-day HDAC inhibitor treatment ± 20nM rapamycin (mean ± SD; * = $p < 0.05$ by two-way ANOVA with Dunnett post-hoc comparison to untreated; n = 3). (**C**) Computational reconstruction of live cell spatial positions upon three-day hydrogel culture of WT ± HDAC inhibitor treatment (5µM SAHA, 5µM SB939, 1µM LBH589). Note that treated and untreated were in separate wells; cells were plotted in the same volume for ease of visualizing relative distances travelled.

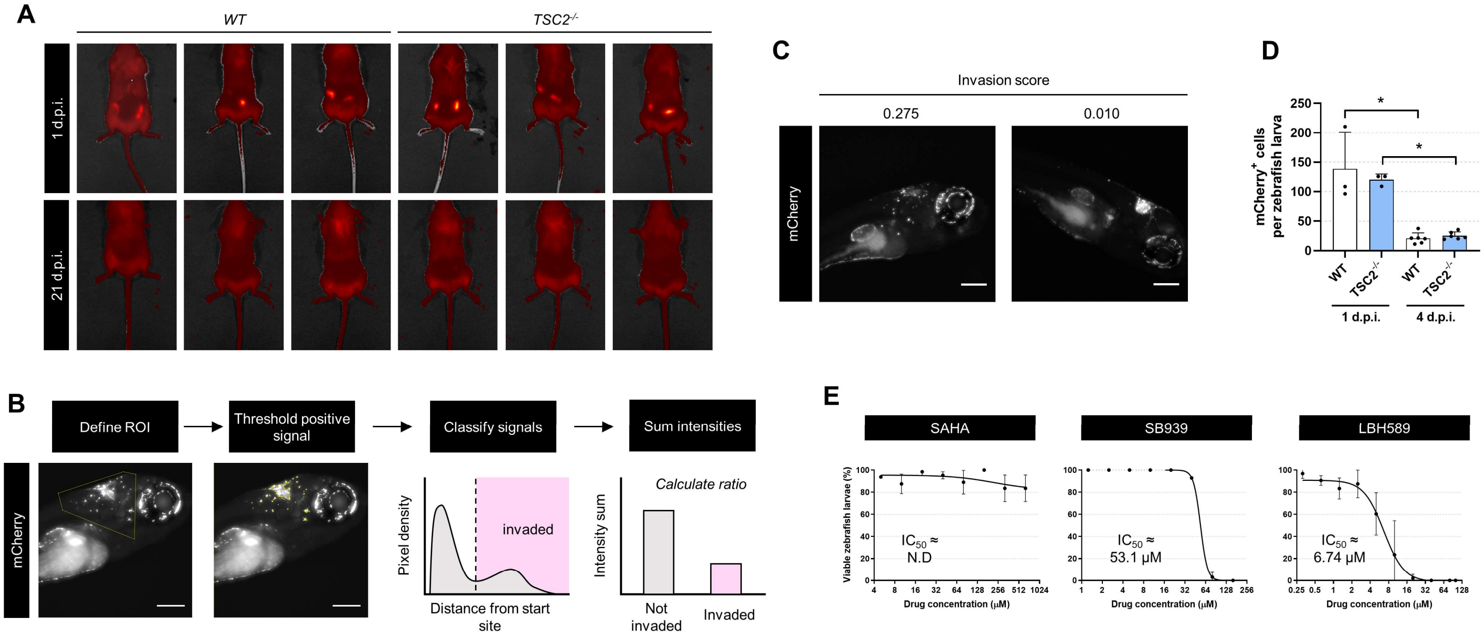

Figure S6. HDAC inhibitors are anti-invasive and selectively cytotoxic towards TSC2$^{-/-}$ cells xenotransplanted into zebrafish. (**A**) Visualization of mCherry^{+} cells by IVIS following subcutaneous transplantation into rear flanks of immunodeficient NSG mice. (**B**) Schematic representation of invasion score calculation in zebrafish larvae. See Supplementary Materials and Methods for more details. Scale bars of 200µm. (**C**) Representative images of *TSC2*$^{-/-}$ mCherry^{+} cells disseminated 4 dpi with the associated invasion score. Scale bars of 200µm. (**D**) Number of mCherry^{+} cells per zebrafish detected by flow cytometry following whole larvae dissociation at 1 and 4 dpi. Each replicate is a pool of 15 – 20 zebrafish larvae (mean ± SD; * = $p < 0.05$ by one-way ANOVA with Bonferroni post-hoc comparisons; n = 3 – 6). (**E**) Dose-toxicity curves of zebrafish larvae treated with HDAC inhibitors by immersion therapy. Data fit via four-parameter logistic regression (mean ± SD; n = 3).

References

[1] S. P. Delaney, L. M. Julian, A. Pietrobon, J. Yockell-Lelièvre, C. Doré, T. T. Wang, V. C. Doyon, A. Raymond, D. A. Patten, A. S. Kristof, M.-E. Harper, H. Sun, W. L. Stanford, *bioRxiv* **2020**, 683359.

[2] R. M. White, A. Sessa, C. Burke, T. Bowman, J. LeBlanc, C. Ceol, C. Bourque, M. Dovey, W. Goessling, C. E. Burns, L. I. Zon, *Cell Stem Cell* **2008**, *2*, 183.

[3] A. Subramanian, P. Tamayo, V. K. Mootha, S. Mukherjee, B. L. Ebert, M. A. Gillette, A. Paulovich, S. L. Pomeroy, T. R. Golub, E. S. Lander, J. P. Mesirov, *PNAS* **2005**, *102*, 15545.

[4] L. M. Julian, S. P. Delaney, Y. Wang, A. A. Goldberg, C. Doré, J. Yockell-Lelièvre, R. Y. Tam, K. Giannikou, F. McMurray, M. S. Shoichet, M.-E. Harper, E. P. Henske, D. J. Kwiatkowski, T. N. Darling, J. Moss, A. S. Kristof, W. L. Stanford, *Cancer Res* **2017**, *77*, 5491.

[5] R. Y. Tam, J. Yockell-Lelièvre, L. J. Smith, L. M. Julian, A. E. G. Baker, C. Choey, M. S. Hasim, J. Dimitroulakos, W. L. Stanford, M. S. Shoichet, *Advanced Materials* **2019**, *31*, 1806214.

[6] D. P. Corkery, G. Dellaire, J. N. Berman, *British Journal of Haematology* **2011**, *153*, 786.

[7] H. M, T. C, S. Wl, M. P, *Angiogenesis* **2006**, *9*, 139.
